# Establishment of a chemokine-based prognostic model and identification of CXCL10+ M1 macrophages as predictors of neoadjuvant therapy efficacy in colorectal cancer

**DOI:** 10.3389/fimmu.2024.1400722

**Published:** 2024-08-07

**Authors:** Abudumaimaitijiang Tuersun, Jianting Huo, Zeping Lv, Yuchen Zhang, Fangqian Chen, Jingkun Zhao, Wenqing Feng, Zhuoqing Xu, Zhihai Mao, Pei Xue, Aiguo Lu

**Affiliations:** ^1^ Department of General Surgery, Ruijin Hospital, Shanghai Jiaotong University School of Medicine, Shanghai, China; ^2^ Shanghai Minimally Invasive Surgery Center, Ruijin Hospital, Shanghai Jiaotong University School of Medicine, Shanghai, China; ^3^ Department of General Surgery, Second People’s Hospital, Kashi, Xinjiang Uygur Autonomous Region, China

**Keywords:** colorectal cancer, neoadjuvant therapy, chemokines, cancer immunity, tumor microenvironment, prognosis

## Abstract

**Background:**

Although neoadjuvant therapy has brought numerous benefits to patients, not all patients can benefit from it. Chemokines play a crucial role in the tumor microenvironment and are closely associated with the prognosis and treatment of colorectal cancer. Therefore, constructing a prognostic model based on chemokines will help risk stratification and providing a reference for the personalized treatment.

**Methods:**

Employing LASSO-Cox predictive modeling, a chemokine-based prognostic model was formulated, harnessing the data from TCGA and GEO databases. Then, our exploration focused on the correlation between the chemokine signature and elements such as the immune landscape, somatic mutations, copy number variations, and drug sensitivity. CXCL10+M1 macrophages identified via scRNA-seq. Monocle2 showed cell pseudotime trajectories, CellChat characterized intercellular communication. CytoTRACE analyzed neoadjuvant therapy stemness, SCENIC detected cell type-specific regulation. Lastly, validation was performed through multiplex immunofluorescence experiments.

**Results:**

A model based on 15 chemokines was constructed and validated. High-risk scores correlated with poorer prognosis and advanced TNM and clinical stages. Individuals presenting elevated risk scores demonstrated an increased propensity towards the development of chemotherapy resistance. Subsequent scRNA-seq data analysis indicated that patients with higher presence of CXCL10+ M1 macrophages in tumor tissues are more likely to benefit from neoadjuvant therapy.

**Conclusion:**

We developed a chemokine-based prognostic model by integrating both single-cell and bulk RNA-seq data. Furthermore, we revealed epithelial cell heterogeneity in neoadjuvant outcomes and identified CXCL10+ M1 macrophages as potential therapy response predictors. These findings could significantly contribute to risk stratification and serve as a key guide for the advancement of personalized therapeutic approaches.

## Introduction

Colorectal cancer (CRC) manifests as one of the most common malignant neoplasms worldwide, holding the third position in terms of incidence and emerging as the second primary source of cancer-induced fatalities globally (1). Estimates predict that the United States will witness 153,020 fresh instances and 52,550 mortalities from colorectal cancer in 2023. Despite continuous advancements in treatment strategies and healthcare standards, the prognosis remains poor for most colorectal cancer patients. This is predominantly a consequence of the late-stage diagnosed that is common among these patients, and the outcomes after radiotherapy and chemotherapy are still far from satisfactory (2). The primary therapeutic approach for CRC is surgical resection. Nevertheless, the effectiveness of solely relying on surgical intervention is not fully satisfactory. Some patients are unable to preserve the anus, and there is a high risk of postoperative recurrence, which adversely affects their quality of life. Preoperative neoadjuvant chemoradiotherapy (nCRT) can effectively downstage the tumor, enhance the rate of curative resection, and improve the postoperative anal preservation rate (3). Nevertheless, nCRT efficacy in improving long-term survival rates is limited, and it may induce postoperative complications and long-term toxicity. Additionally, roughly one in five patients with locally advanced colorectal cancer may demonstrate a less than satisfactory response to nCRT, potentially missing the optimal opportunity for curative surgery. Consequently, future research should focus on integrating multiple biomarkers to develop predictive models, aiming to enhance prognosis and treatment outcomes (4). Therefore, a profound understanding of tumor characteristics and the identification of reliable prognostic markers are crucial for providing personalized treatment approaches aimed at elevating the life expectancy of individuals diagnosed with colorectal cancer.

Chemokines, small molecules of 8–12 kDa, are categorized into four primary subclasses: C, CC, CXC, and CX3C, based on the location of their N-terminal cysteine residues. To date, fifty distinct chemokine ligands and their nineteen matching receptors are recognized in the human system. Within oncological processes, chemokine signaling and the chemotaxis of diverse cellular entities is pivotal for sculpting the tumor microenvironment (TME), significantly impacting tumor progression, metastasis, and the establishment of immune responses via the mobilization and stimulation of inherent effector cells (5, 6). Chemokines significantly contribute to cancer therapy, especially in enhancing the effectiveness of checkpoint blockade therapy, Methods that propel the manifestation of chemokines attracting T cells, or counteract the chemokine inhibitory trajectory, which is responsible for the mobilization of myeloid-derived suppressor cells and regulatory T cells, possess the capability to boost the receptiveness of immunotherapy in immunologically inactive and morphologically altered tumors (7). Certain antagonists targeting chemokine receptors have been validated as potential therapeutic targets in preclinical research pertaining to colorectal cancer. For instance, in phase I trial revealed that the CXCR4 inhibitor exhibited clinically safe and well-tolerated antitumor activity in CRC and other solid tumors, with a primary response rate of 20% of stable disease (8). In a different phase I study, employing CCR5 antagonists for managing advanced resistant CRC with liver metastases exhibited anti-tumor effectiveness (9). Nonetheless, an exhaustive assessment of chemokines’ influence on the prognosis and effectiveness of neoadjuvant treatment for CRC remains inadequate.

In our research, we constructed a chemokine-based prognostic model. Subsequently, we demonstrated that a chemokine-related model contributes to distinguishing the prognosis, immune infiltration, and drug sensitivity of CRC patients. Furthermore, we explored the heterogeneity between neoadjuvant therapy efficacy and identified CXCL10+M1 macrophages as potential biomarkers for predicting neoadjuvant therapy efficacy.

## Materials and methods

### Data collection

As the train group, raw bulk transcriptome counts data and clinicopathological information of colon adenocarcinoma (COAD) and rectum adenocarcinoma (READ) were obtained from the University of California Santa Cruz (UCSC, https://xenabrowser.net/datapages/), with the TCGA cohort comprising a merged dataset of COAD and READ. The training set included data from 581 CRC patients sourced from The Cancer Genome Atlas (TCGA), featuring comprehensive clinicopathological profiles and exhaustive follow-up data. Subsequently, ensembl IDs were converted to official gene symbols, and the data underwent normalization and log2 transformation. As the test group, we retrieved survival outcomes and gene expression profiles for an independent cohort of 556 CRC cases from the Gene Expression Omnibus (GEO) databases (https://www.ncbi.nlm.nih.gov/geo). Besides, GSE45404 (Affymetrix Human Genome U133 Plus 2.0 Array) datasets were applied to analyze the neoadjuvant chemoradiotherapy response of locally advanced rectal cancer. Somatic mutation profiles and copy number alteration data were acquired through the application of the R package “TCGAbiolinks”. ScRNA-seq data for tumors and adjacent normal tissues from 10 CRC patients were collected from dataset GSE146771 (Smart-seq2). In addition, scRNA-seq data of tumor tissue from 13 pathological complete response (pCR) and 4 Non pathological complete response (NpCR) patients after neoadjuvant immunotherapy for colorectal cancer were collected from the GSE205506 dataset. The baseline characteristics of CRC patients in the TCGA and GSE39582 datasets were detailed in **Supplementary Table S1.**

### Identification of differentially expressed prognostic chemokines

Differentially expressed genes (DEGs) between CRC samples and normal colorectal samples (P value< 0.05, |logFC| ≥ 1) were obtained using the “limma” R package. official gene symbols name was used to screen for differentially expressed prognostic chemokines (DEPCs). Prognostic chemokines were identified by conducting univariate Cox regression analysis using the “survival” R package; these selected chemokines were identified as candidate DEPCs. which were screened again based on the LASSO regression to preclude model overfitting. The optimal penalty coefficient lambda value was determined via 10-fold cross-validation using the “glmnet” R package. The risk score for each patient was computed using the formula: $\text{Risk score}=\sum_{i=1}^{n}\left(exp_{i}\times coef_{i}\right)$ , In this formula, $exp_{i}$ denotes the expression level of each chemokine, while $coef_{i}$ corresponds to its associated coefficient. Subsequently, the risk score signature was applied to validation cohort, GSE39582 cohort as well as the neoadjuvant chemoradiotherapy GSE45404.

### Construction and validation of prognostic model

Univariate and multivariate Cox regression analyses were conducted to confirm the independent prognostic factor of the risk score. The forest plot unveiled the outcomes. Kaplan-Meier curves were constructed using the “survminer” R package, and the log-rank test was utilized to differentiate overall survival (OS), progression-free survival (PFS), or disease-free survival (DFS) outcomes among high-risk and low-risk groups.

We utilized the “RColorBrewer” software in accordance with the solid tumor immune classification developed by Thorsson et al., which includes wound healing (C1), IFN-γ dominance (C2), inflammation (C3), lymphocyte depletion (C4), immune silencing (C5), and TGF-β dominance (C6) (10). Subsequently, we compared the differences in immune classifications among different risk groups.

A prognostic nomogram was constructed based on Clinical features including age, T stage and risk score, utilizing multivariable Cox and stepwise regression analyses. The “regplot” package facilitated the display of the nomogram plot. Calibration plots and Decision Curve Analysis (DCA) were employed to assess the clinical applicability of the model. Furthermore, The R package “timeROC” was employed to carry out time-dependent receiver operating characteristic (ROC) curve analysis, with the aim of identifying the specificity and sensitivity of the risk signature for survival rates at intervals of 1, 3, and 5 years.

The “surv_cutpoint” algorithm from the “survival” R package was utilized to identify the ideal cut-off value for the risk score. Patients were subsequently divided into two categories - low risk and high risk - according to this optimal cut-off value.

### Functional enrichment analysis

Utilizing the “limma” R package, differentially expressed genes between the high-risk and low-risk categories were identified (|log FC|>0.585, P<0.05). Subsequently, the ‘clusterProfiler’ R package facilitated functional annotation through Gene Ontology (GO) and Kyoto Encyclopedia of Genes and Genomes (KEGG).

### Gene set enrichment analysis

In order to investigate the molecular and biological distinctions between the two categories, The “c2.cp.kegg.v2023.1.Hs.symbols.gmt” and “h.all.v2023.1.Hs.symbols.gmt” gene sets from the molecular signature database were utilized as the reference in the conduct of Gene Set Enrichment Analysis (GSEA). We considered pathways to reach statistical significance when exhibiting a normalized P value less than 0.05 coupled with a false-discovery rate (FDR) q value under 0.25. The top enriched pathways were determined by ranking the normalized enrichment scores (NESs). We conducted gene set variation analysis utilizing the GSVA package (version 1.46.0). Differential pathway activity between the non-pathological complete response (NpCR) and pathological complete response (pCR) groups was quantified employing the limma package (version 3.54.2). In addition, the standard procedure of the “AUCell” R package is used to explore the pathway activity of individual cells.

### Evaluation of immune cell infiltration

The disparity in immune cell infiltration between the two categories was assessed via the CIBERSORT algorithm. This tool utilizes expression data to depict cell composition in complex tissues from preprocessed gene expression profiles (11). CIBERSORT’s LM22 delineates 22 immune cell subsets, accessible from the CIBERSORT web portal.

### Somatic mutation and copy number variation analysis

Utilization of the “Maftools” R package facilitated the analysis of gene mutations across different risk subgroups. Subsequently, the exploration of amplifications and deletions with CNV data was enabled by GISTIC2.0.

### Drug sensitivity prediction

Harnessing resources from Genomics of Drug Sensitivity in Cancer (GDSC) pharmacogenomics repository, an examination was conducted on chemotherapeutic response profiles of patients within TCGA dataset. Calculation of half-maximal inhibitory concentration (IC50) was achieved through “oncoPredict” R package. Specifically, we focused on several drugs commonly employed in colorectal cancer neoadjuvant chemotherapy and explored the variations in sensitivity observed between categories of low risk and high risk.

### Consensus clustering analysis

Based on the chemokine model, the k-means approach was employed to discern and categorize the patient into molecular subtypes. The determination of cluster quantity and their robustness was conducted using the “ConsensusClusterPlus” package, with 50 iterations to confirm the stability of the subtyping. Subsequently, an unsupervised clustering algorithm served to categorize patients into varied subtype classifications (cluster A, cluster B, and cluster C) based on the expression of prognostic DEGs for further analysis.

To assess immune cell infiltration and the and clinical value of each consensus clustering subtype, single-sample gene set enrichment analysis (ssGSEA) and Kaplan-Meier survival analysis were performed.

### Analysis of neoadjuvant therapy efficacy

For each individual within the GSE45404 cohort, a risk score was computed. Following the median risk score, patients were subsequently partitioned into either low- or high-risk categories. Then we compared the proportion of response (TRG 1 and 2) versus Non-response (TRG 3, 4 and 5) between low - and high -risk groups, CIBERSORT and ssGSEA were utilized to quantify the immune cell infiltration of each sample. Subsequently, a comparison was conducted to evaluate the disparities in immune cell infiltration and expression of Model-based chemokine signatures between the response and Non-response groups.

### Single−cell RNA sequencing analysis

Analysis of the scRNA data was performed utilizing Seurat (v4.3.0.1) (12). For the GSE146771 (Smart-seq2 platform) datasets (13) individual cells with gene counts exceeding 7000 or falling below 700 were excluded, Additionally, cells exhibiting over 10% of mitochondria-derived UMI counts and erythrocyte-derived UMI counts surpassing 20% were considered of suboptimal quality and subsequently excluded. For the GSE205506(10X Genomics) dataset individual cells with gene counts exceeding 5000 or falling below 500 or genes per cell identified or those possessing over 20% of mitochondria-derived UMI counts were evaluated as subpar in quality, leading to their subsequent removal. Initial data normalization was performed using the ‘NormalizeData” and “ScaleData” routines. This was followed by the identification of the 2000 most variable genes through the “FindVariableFeatures” function. Dimensionality reduction was achieved via principal component analysis (PCA) leveraging these variable genes, with a resolution parameter set at 0.8. For the GSE205506(10X Genomics) dataset (14) set the resolution parameter to 0.4. To mitigate batch-related variabilities, an integration of datasets across different experimental conditions was conducted using the “RunHarmony” function. Marker gene candidates were ascertained utilizing the “FindAllMarkers” function. Annotation of diverse cellular populations leveraged the expression profiles of established marker genes as referential benchmarks. Visualization of the cellular landscape via the tSNE algorithm.

### Pseudotime trajectory analysis

For the elucidation of cell differentiation trajectories, Monocle2 (version 2.26.0) was employed to conduct pseudotime analyses (15). For the trajectory analysis, genes exhibiting a mean expression level exceeding 0.1 were selectively included, This was followed by the application of the differentialGeneTest() function, which served to identify and exclude genes for cell ordering purposes, adhering to a stringent q-value threshold of less than 0.01. Dimensionality reduction was performed utilizing the reduceDimension() function, which integrates the DDRTree algorithm. All analytical procedures were executed using the functions” default parameters. The final cell ordering was accomplished through the “orderCells” function.

### Intercellular communication

The intercellular communication analysis was carried out with the assistance of the “CellChat”(1.6.1) R package, this tool capitalizes on established knowledge of signaling ligand-receptor interactions and their related cofactors, facilitating the prediction of potential intercellular communication networks derived from scRNA-seq data (16). Each group was subjected to individual analysis, with the “mergeCellChat” function being used to evaluate the distinctions existing among the two categories. The receptor-ligand interaction database “CellChatDB.human” was selected, and default parameters were employed.

### Predicting differentiation potential with CytoTRACE

The validation of the CytoTRACE algorithm, conducted by Gulati et al., utilized extensive datasets to demonstrate its superiority over existing computational methods for cells differentiation. This algorithm effectively identifies predictive factors for cell differentiation status, independent of tissue type, species, or platform (17). In the assessment of differentiation potential within malignant cell populations, the R package CytoTRACE (version 0.3.3) was deployed to derive CytoTRACE scores. The CytoTRACE scoring system is scaled from 0 to 1, with elevated scores denoting an augmented propensity for cellular differentiation. We further applied CytoTRACE to epithelial cells, endothelial cells, cancer-associated fibroblasts, and fibroblast subsets, and compared their differentiation potential between the pCR and NpCR groups.

### Cell-type-specific regulon analysis

The enrichment of pivotal transcriptomic factors in macrophage clusters was examined using single-cell regulatory network inference and clustering [SCENIC (1.3.1)]. Leveraging the SCENIC workflow (18), Genes that were manifested in no less than 3% of the samples and cells exhibiting more than 0 unique molecular identifiers were retained for further analysis. Following this, the expression levels were normalized by log2(filtered expr+1). Subsequently, GENIE3 was used to identify the target genes potentially regulated by transcription factors (TFs). “RcisTarget” was employed to perform DNA-motif enrichment analyses, enabling the detection of direct binding sites (regulons). AUCell (1.22.0) was applied for the assessment of individual regulon activities within single cells.

### Tissue microarray and patients and follow-up

In this study, for the purpose of validation analysis, we included two distinct cohorts of patients with CRC from Ruijin Hospital, Shanghai, China. The cohort 1 consisted of 70 patients whose tissue samples were collected from surgeries performed between 2010 and 2011, with follow-up data gathered bi-monthly through outpatient visits or telephone communications. Inclusion criteria encompassed a pathological diagnosis of colorectal cancer, no preoperative radiotherapy or chemotherapy, and no history of other malignancies. The collection of follow-up data was completed by August 2015. The cohort 2 included 72 patients, with tissue samples obtained from surgeries conducted between July 2020 and October 2023. The evaluation of tumor regression grading conformed to the criteria established in the 8th edition of the American Joint Committee on Cancer’s Tumor Regression Grading system, utilizing pathological assessments of postoperative tissue samples. Criteria for inclusion required a pathological diagnosis of colorectal cancer, receipt of neoadjuvant therapy before surgery, and no history of other malignancies.

### Fluorescence-based multiplex immunohistochemistry staining

The study followed a standard protocol for tissue sectioning and immunohistochemical analysis. Tumor tissues and adjacent normal tissues were fixed in formalin for 24 to 48 hours starting within 5 minutes post-excision. and then deparaffinized in xylene and ethanol gradients to obtain 4μm thick sections. The process of antigen retrieval was carried out in a high-pressure heat repair procedure utilizing citrate buffer at pH 6.0. Following the blocking of endogenous peroxidase with 3% hydrogen peroxide, the tissue sections were pre-incubated with 10% normal goat serum and left overnight for incubation with primary antibodies, subsequently followed by the appropriate HRP-conjugated secondary antibodies for 20 minutes at ambient temperature and a final step of diaminobenzidine staining. The primary antibodies used in this study were as follows: CXCL-10 (Proteintech Group, 10937–1-AP), CD163(Abcam, MA, ab182423), and CD68 (Cell Signaling, 76437T). The secondary antibody was (Sanying Biotech, SA00013–2) and counterstained with DAPI. The quantitative analysis was performed by two independent pathologists, who randomly captured 5 images at 60x magnification from each IHC-stained slide for scoring. The scores were then normalized after averaging and log2 transformation, followed by visualization and statistical analysis using R software.

### Statistical analysis

Statistical computation and data visualization were carried out using R (v4.2.0). Chi-square testing assessed the association between CRC patient clinicopathological traits and risk categories. Survival outcomes were analyzed via Kaplan-Meier estimates. Cox regression models, both univariate and multivariate, pinpointed independent prognostic indicators. Variable differences across risk cohorts were evaluated with Wilcoxon’s test, and ANOVA facilitated multi-group comparisons. Significance was set at P< 0.05. All methodologies adhered to relevant guidelines and regulations.

## Results

### Identification of differentially expressed prognostic chemokines

**Figure 1:**
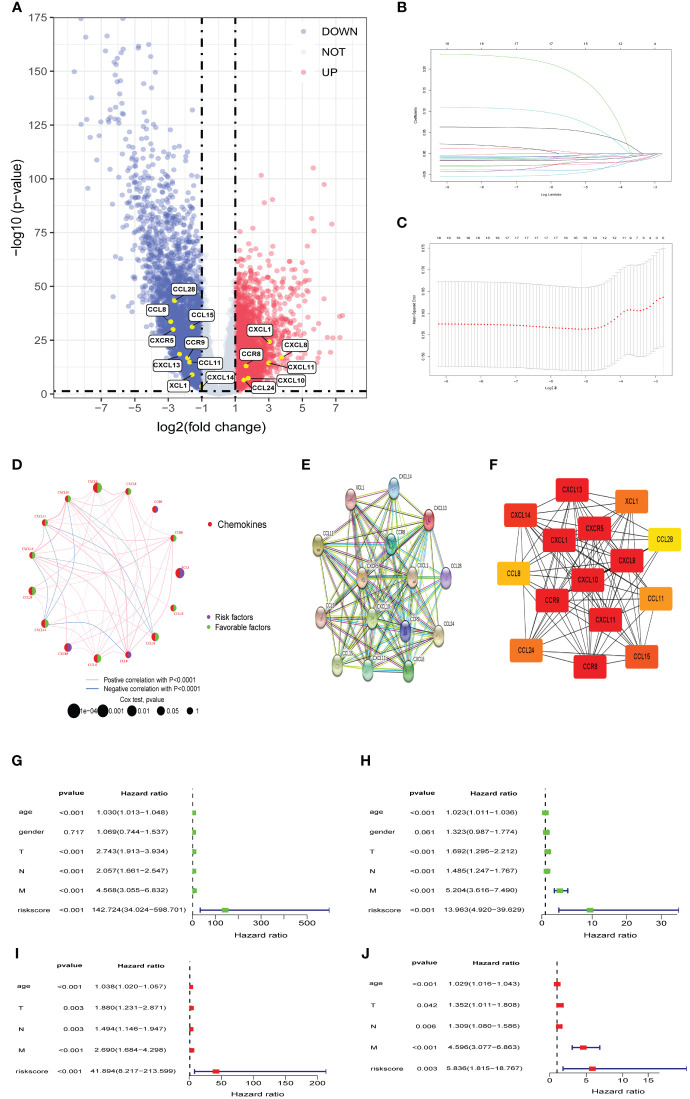
Identifification of differentially expressed prognostic chemokines **(A)** Volcano plot of DEPCs between Tumor and Normal. Blue and red dots denote genes with upregulation in Normal and Tumor tissues, respectively. **(B, C)** The cvfit and lambda trace plots illustrate the execution of LASSO regression adhering to the minimum criteria. **(D)** A network graphically represents the interplay among selected chemokines. Connective lines between elected chemokines reveal interactions, with line thickness indicating association strength. Colors blue and pink differentiate between negative and positive correlations, respectively. **(E)** A PPI network of DEPCs. **(F)** Hub genes identifited by betweenness centrality according to cytoHubba plug-in. **(G, I)** Outcomes from univariate and multivariate Cox regression assessments of risk scores and clinicopathological attributes for OS within the TCGA cohort. **(H, J)** Outcomes from univariate and multivariate Cox regression assessments of risk scores and clinicopathological attributes for OS within GSE39582 cohort. PPI: protein-protein interaction.

In the TCGA cohort, we identified a total of 7632 DEGs between CRC samples and normal colorectal samples, adhering to a defined criterion of p value was less than 0.05 and an absolute log2 fold change greater than 1. Out of these, 3703 genes exhibited upregulation, whereas 3,929 genes were downregulated in CRC tissues. In order to create a predictive model, we performed LASSO Cox regression analysis using the expression of the DEPCs. we successfully filtered out 15 chemokines using the optimal value of lambda (λ), **Figure 1A** presents a volcano plot illustrating the distribution of differentially expressed genes, **Figure 1B** provides depiction of the trajectory of these expression alterations as informed by the LASSO regression analysis. **Figure 1C** shows the confidence interval for each lambda value. In addition, a network was constructed to display the overall landscape of the connections among the chosen chemokines, regulatory links, and prognostic significance in patients with CRC (**Figure 1D**). To explore the interactions among selected chemokines, PPI analysis was conducted to elucidate the molecular interplay, and operationalized by setting the minimum interaction score at a high-confidence level of 0.9. (**Figure 1E**). and then interaction information of the PPI network imported to Cytoscape for visualization. Finally, Utilizing the cytoHubba algorithm, our investigation identified CXCL10, CXCL11, CXCL8, CXCL1, CXCL13, CXCL14, CXCR5, CCR9, and CCR8 as hub genes (**Figure 1F**). Additionally, Cox univariate and multivariate regressions were conducted to assess the prognostic independence of the signature. univariate analysis corroborated a significant correlation between the signature’s risk score and OS in CRC patients within the TCGA cohort. (HR=142.724, p< 0.001; **Figure 1G**) and GSE39582 cohort (HR=13.963, p< 0.001; **Figure 1H**). Subsequent multivariate Cox regression analysis corroborated the risk score as an independent prognostic factor for OS in the TCGA cohort (HR=41.894, p< 0.001; **Figure 1I**) and GSE39582 cohort (HR=5.836, p =0.003; **Figure 1J**).

### Assessment of chemokine related prognostic model

**Figure 2:**
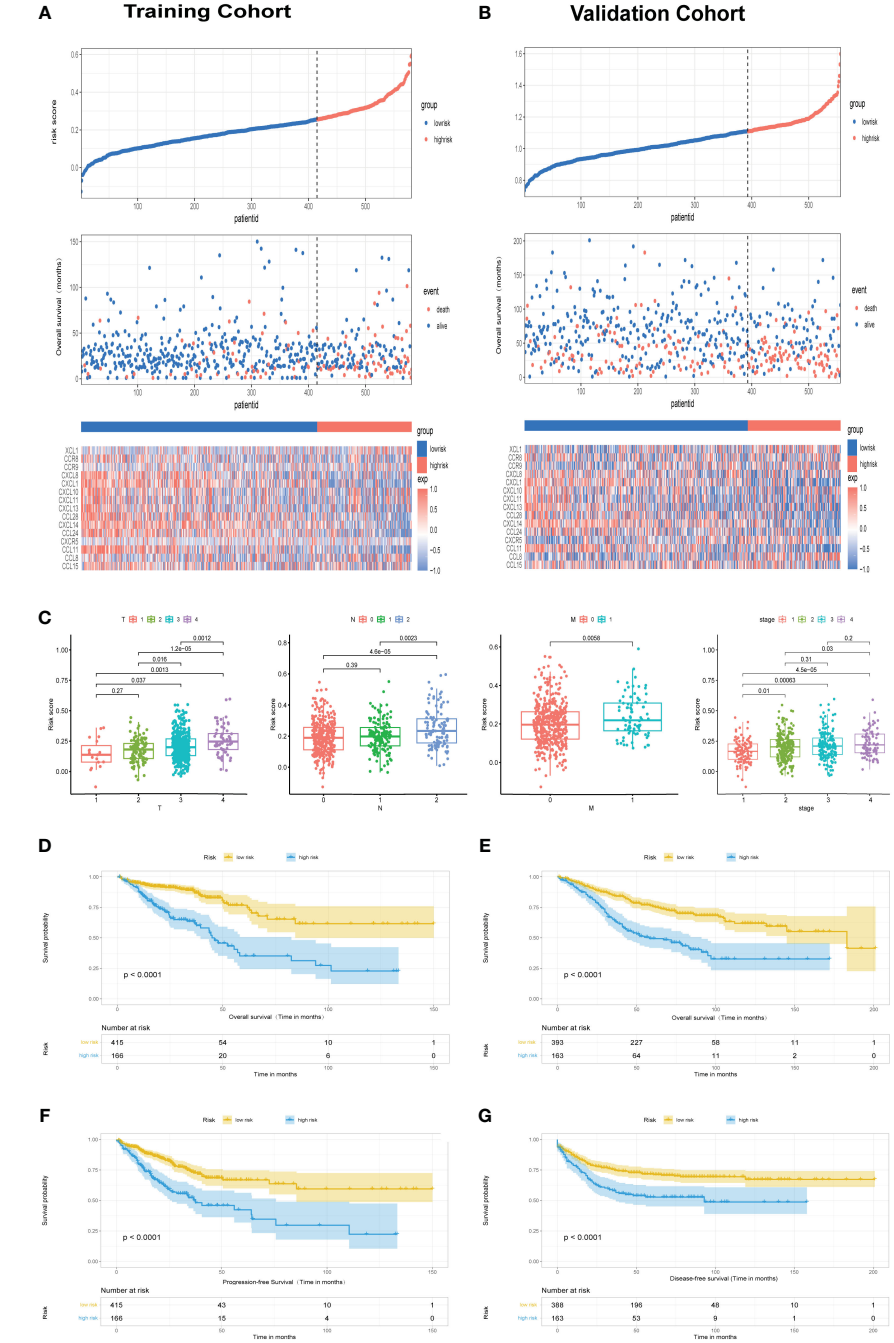
Assessment of chemokine Related Prognostic Model. **(A, B)** Risk score and corresponding overall survival outcomes in TCGA and GSE39582 cohorts, respectively. **(C)** Relationship of risk score with clinicopathological features attributes such as T, N, M, along with clinical stage. **(D, E)** Kaplan-Meier survival plots delineate OS among high- and low-risk groups in TCGA cohort and GSE39582 cohort, respectively. **(F)** Kaplan-Meier survival plots delineate PFS among high- and low-risk groups in TCGA cohort. **(G)** Kaplan-Meier survival plots delineate DFS among high- and low-risk groups in GSE39582 cohort.

Patients were stratified into low- and high-risk categories according to the optimal cutoff in each cohort, Elevated fatality incidences in the high-risk categories denoted poorer outcomes in both TCGA cohort (**Figure 2A**) and GSE39582 cohorts (**Figure 2B**). Disparities in risk scores were assessed among clinical-pathological categories, revealing significant variance with T, N, M, and clinical stages. Higher stages paralleled increased risk scores (**Figure 2C**). Significant survival discrepancies were observed, with high-risk individuals demonstrating notably reduced overall survival within both TCGA cohort (**Figure 2D**, P<0.0001) and GSE39582 cohort (**Figure 2E**, P<0.0001), aligning with Progression-Free Survival (PFS) in the TCGA cohort (**Figure 2F**, P<0.0001) and DFS in GSE39582 cohort (**Figure 2G**, P<0.0001).

**Figure 3:**
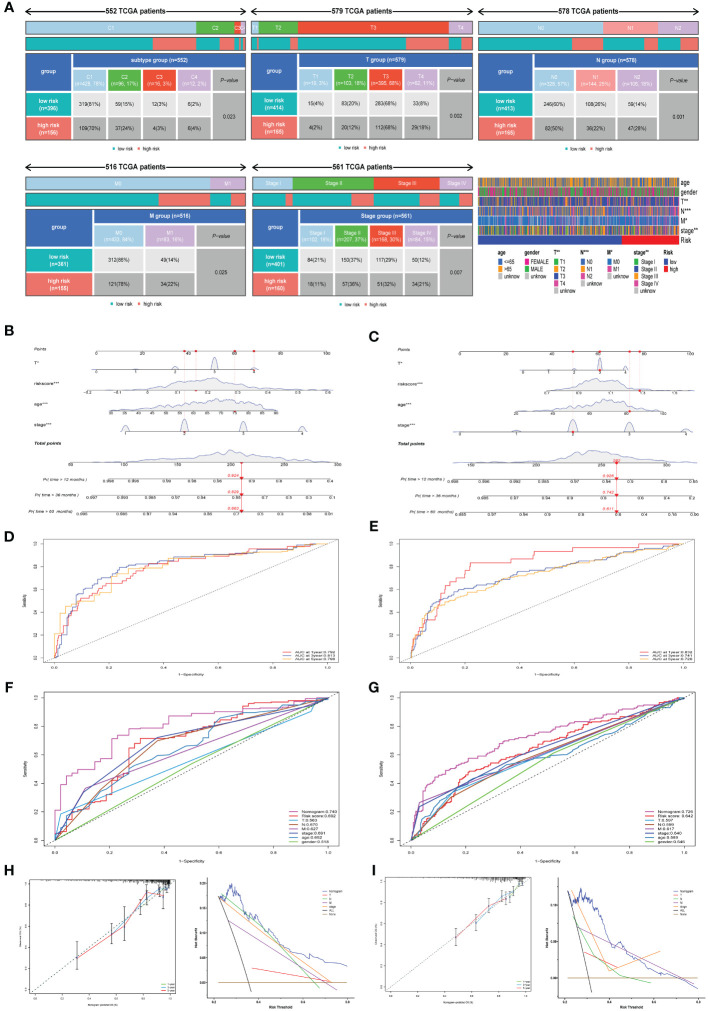
Assessment of chemokine Related Prognostic Model. **(A)** Heatmap and table illustrate the variance in immune subtypes and diverse clinicopathological features between two categories. **(B, C)** Nomograms forecasting 1-, 3-, and 5-year overall survival probabilities within TCGA and GSE39582 cohorts. **(D, E)** ROC analysis illustrates the nomogram’s efficacy in forecasting 12-, 36-, and 60-month overall survival across TCGA and GSE39582 cohorts. **(F, G)** Comparison of prognostic accuracies via ROC for nomogram, risk score and other variables in TCGA and GSE39582 cohorts. **(H, I)** Calibration plots and decision curve analysis for the nomogram are illustrated for both TCGA and GSE39582 cohorts. *P<0.05; **P<0.01; ***P<0.001.

Exploration of clinicopathological variables within TCGA cohorts uncovered substantial differences in TNM classifications and clinical stage across risk categories (**Figure 3A**, p< 0.05). Immune subtypes also demonstrated a significant association with risk categories. Elevated risk corresponded to more severe disease progression, highlighting the predictive capacity of chemokine-based risk scores for colorectal cancer prognosis. Utilizing multivariate Cox regression, a nomogram incorporating age, T stage, clinical stage, and risk score was constructed to predict 1-, 3-, and 5-year survival probabilities in TCGA (**Figure 3B**) and GSE39582 cohorts (**Figure 3C**). Time-dependent ROC curves and corresponding AUC values substantiated the risk score’s predictive accuracy for OS in TCGA cohort (AUC of 1-year = 0.792; AUC of 3-year =0.813; AUC of 5-year = 0.798; **Figure 3D**), and GSE39582 cohort(AUC of 1-year = 0.832; AUC of 3-year =0.741; AUC of 5-year = 0.726; **Figure 3E**).Time-dependent ROC curve corroborated the nomogram’s superior prognostic precision relative to traditional clinicopathological features in TCGA cohort (**Figure 3F**) and GSE39582 cohort (**Figure 3G**). Calibration assessments affirmed model precision for predicting 1-, 3-, and 5-year survivals. Comparative decision curve analysis (DCA) underscored the nomogram’s enhanced predictive capacity over other markers within TCGA cohort (**Figure 3H**) and GSE39582 cohort (**Figure 3I**), underscoring its prognostic utility in clinical practice.

### Potential mechanism analysis of chemokine-related gene signature

**Figure 4:**
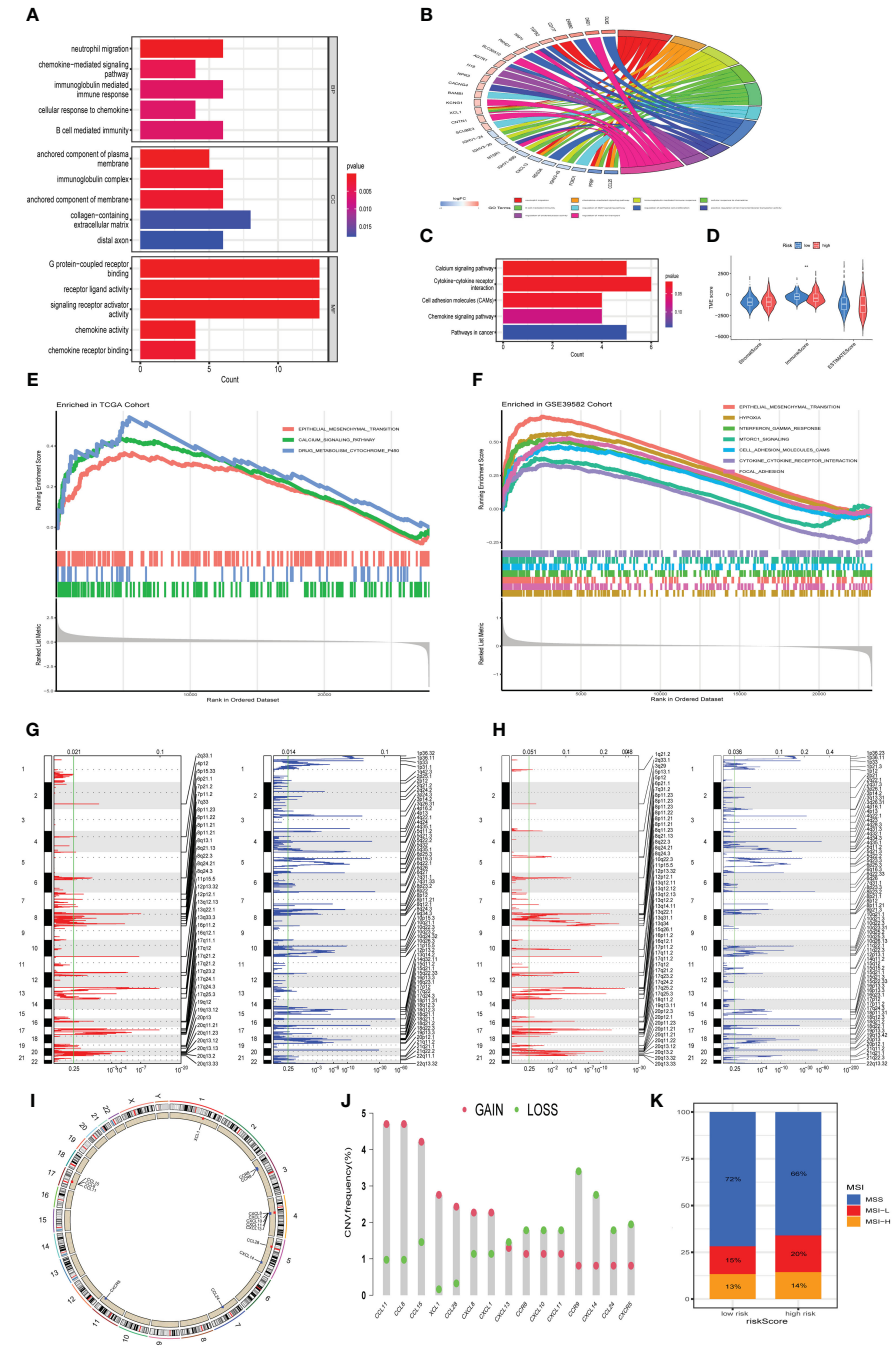
Potential mechanism analysis of chemokine-related gene signature. **(A, B)** Bar and Circos plots illustrate the GO enrichment analysis for DEGs, contrasting the high-risk with the low-risk categories in the TCGA cohort. **(C)** Bar graph representation of KEGG enrichment analysis comparing DEGs within high-risk versus low-risk categories in the TCGA cohort. **(D)** Associations of risk score with immune and stromal scores within the TCGA cohort. **(E, F)** Gene sets enriched in high-risk categories in TCGA cohort and GSE39582 cohort, respectively. (P< 0.05, FDR< 0.25). **(G)** Amplifcation and deletion regions detected in the high-risk categories in TCGA cohort. **(H)** Amplifcation and deletion regions detected in the low risk group in TCGA cohort. **(I)** Locations of CNV alterations in chemokine-related genes on 23 chromosomes. **(J)** Incidence of CNV gain, loss, and non-CNV among chemokine-related genes. **(K)** The distribution of microsatellite status across high-risk and low-risk classifications within the TCGA cohort. **p< 0.01.

To elucidate the underlying mechanisms contributing to the disparate outcomes in high-risk versus low-risk categories, GO, KEGG, and GSEA analyses were conducted. DEGs were identified with a threshold of log2|FC| > 0.585 and P< 0.05. GO enrichment analysis showed DEGs prominently associated with chemokine-mediated signaling pathway, immunoglobulin-mediated immune response, and B cell mediated immunity (**Figures 4A, B**). Furthermore, KEGG pathway analysis revealed notable enrichments in the Calcium signaling pathway, Cytokine-cytokine receptor interaction, and Cell adhesion molecules (**Figure 4C**). The ESTIMATE analysis showed reduced immune scoring in the high-risk categories versus the low-risk categories (**Figure 4D**). Furthermore, GSEA demonstrated significant enrichment of Epithelial Mesenchymal Transition (EMT) gene sets within the TCGA Cohort. (**Figure 4E**). Similarly, the GSE39582 Cohort gene sets were enriched in EMT, hypoxia, interferon gamma response, mtorc1 signaling, and cytokine-cytokine receptor interaction pathway (**Figure 4F**). In conclusion, our findings suggest that the chemokine-related risk score signature is primarily associated with tumor metastasis, tumor immunity, and drug resistance in colorectal cancer. The GISTIC2.0 results indicated an elevated CNV rate, mainly deletions, in high-risk patients (**Figure 4G**), in contrast to a lesser CNV rate in the low-risk demographic (**Figure 4H**). The location of CNV of all chemokine-related genes on chromosomes was exhibited in **Figure 4I**. We determined the frequency of CNV in selected chemokine-related genes and most genes have amplification and deletion (**Figure 4J**). Ultimately, we evaluated the microsatellite status distributions across groups delineated by risk level, discovering an increased incidence of microsatellite instability-low (MSI-L) and a diminished incidence of microsatellite stability (MSS) within the high-risk population (**Figure 4K**).

### The mutational landscape of chemokine-related prognostic models in CRC

In order to elucidate potential disparities in gene mutation patterns between the high-risk and low-risk categories, somatic mutation profiling was undertaken. Generally, mutation rates in the high-risk categories were observed to be elevated compared to those in the low-risk categories. Specifically, TP53 (66% vs.57%), SYNE1(33% vs.25%) and MUC16(31% vs.23%) were the most prevalent in high-risk group. (**Supplementary Figures S1A–S1C**). By conducting mutual exclusion and cooperation analysis among mutated genes, Gene mutation synergies were found among most genes in the both high-risk (**Supplementary Figure S1D**) and low risk group (**Supplementary Figure S1E**), notable genes like TTN, SYNE1, and MUC16 were included. Additionally, a substantial mutational mutual exclusion was observed between TP53-MUC16 and TP53-PCLO in the low risk categorized. (**Supplementary Figure S1E**). The distribution of TP53 and SYNE1 mutation sites across the protein structure were shown in (**Supplementary Figure S1F**). Additionally, the correlation of the risk score and TMB was investigated. An elevation in TMB was discerned within the categories deemed high-risk when contrasted with the categories of lower risk, but there was no statistical significance (p= 0.13, **Supplementary Figure S1G**), and we found a weak positive interrelation of risk score with TMB (log 10) (r = 0.1 6, p< 0.05, **Supplementary Figure S1H**).

### Immune cell infiltration characteristics and drug sensitivity analysis in chemokine-related subgroups

**Figure 5:**
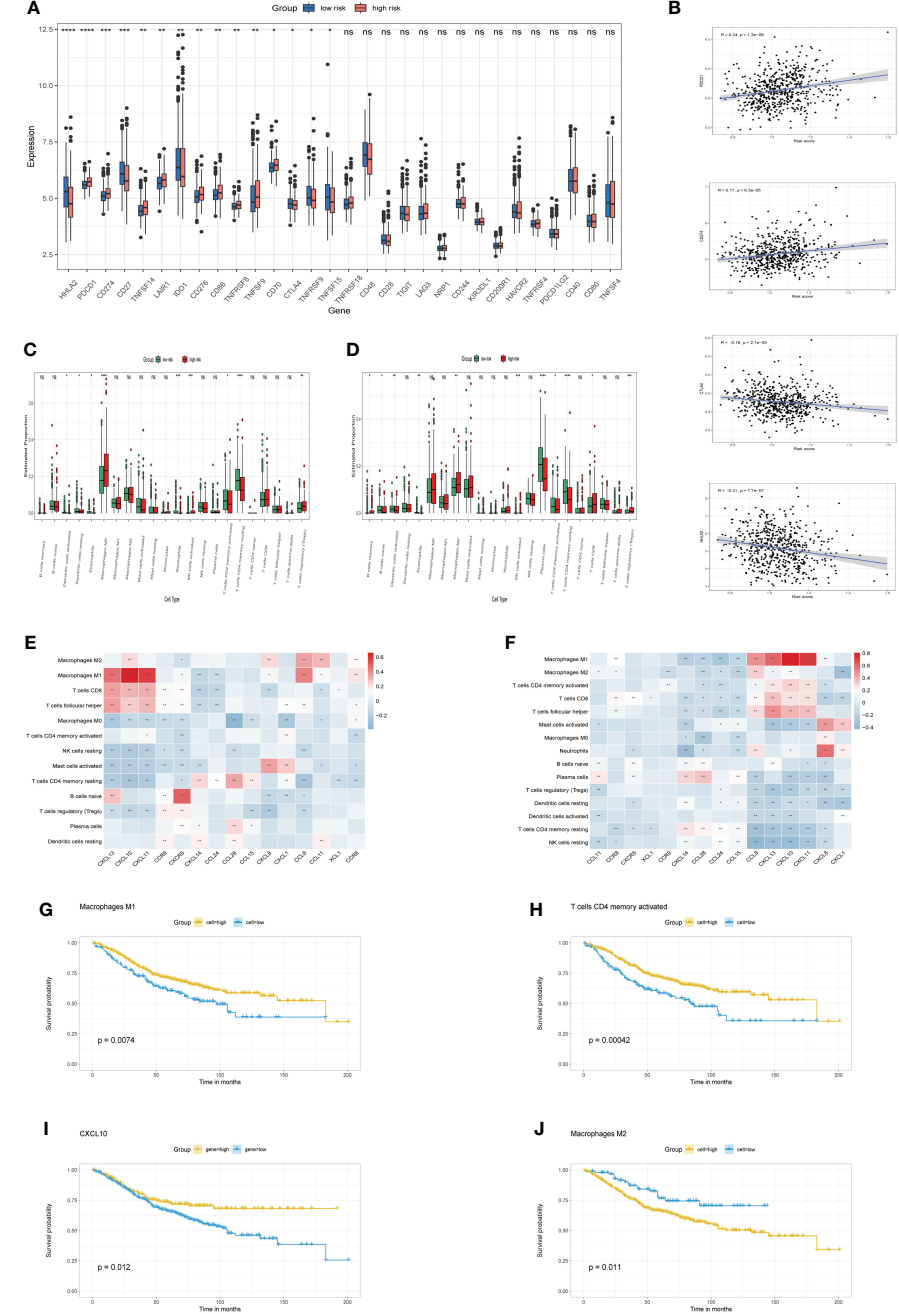
immune checkpoints and Immune cell infiltration characteristics **(A)**. The boxplots illustrate differential expression of immune checkpoint genes in high-risk versus low-risk categories. **(B)** Association of risk score with immune checkpoint markers (PDCD1, CD274, CTLA4, HHLA2). **(C, D)** Boxplots contrast 22 immune cell profiles between high-risk and low-risk groups in TCGA and GSE39582 cohorts. **(E, F)** Correlation between model genes and abundance of immune cells in TCGA cohort and GSE39582 cohort, respectively. **(G-J)** Kaplan-Meier curve of OS for CRC patients grouped by the infiltration or expression levels. *p< 0.05, **p< 0.01, ***p< 0.001, and ****p< 0.0001, ns, No significance.

Expression profiles of immune checkpoint genes were contrasted between high-risk and low-risk categories. Notably, **Figure 5A** depicts substantial disparities in established immunotherapeutic markers, including PDCD1, CD274, CTLA4, and HHLA2 between the two categories. Furthermore, Correlation assessments indicated a robust positive linkage of risk scores with immune-inhibitor molecules, including PDCD1 and CD274, while a negative correlation was observed with CTLA4 and HHLA2 (**Figure 5B**). Moreover, we characterized the immune cell infiltration profiles across individuals in both TCGA and GSE39582 cohorts via the CIBERSORT algorithm. Subsequently, we evaluated the immune cell composition variance between high-risk and low-risk categories within each cohort, revealing elevated levels of M0 and M2 macrophages, along with regulatory T cells, in the high-risk category, while the abundance of activated CD4 memory T cells, resting CD4 memory T cells and plasma cells was elevated in the low-risk group compared with the high-risk group (**Figures 5C, D**). Correlation analysis indicated a robust positive association between CXCL10 expression and M1 macrophage prevalence in both the TCGA and GSE39582 cohorts. (**Figures 5E, F**, p<0.01). Additionally, we found that the infiltrating abundance of M1 macrophages, M2 macrophages, CD4 memory activated T cells and expression levels of CXCL10 was significantly related to OS rate in GSE39582 cohort. Specifically, patients with higher infiltration of M1 macrophages, CD4 memory activated T cells and elevated expression of CXCL10 exhibited a better prognosis (**Figures 5G–I**, p<0.05), while patients with higher infiltration of M2 macrophages had a worse prognosis (**Figure 5J**, p<0.05). Furthermore, our research demonstrated a direct correlation between the risk score and the prevalence of M2 macrophages (**Supplementary Figure S2A**, p<0.05), alongside an inverse correlation with the occurrence of plasma cells. (**Supplementary Figure S2B**, p<0.05).

To probe the differential drug resistance across risk categories, IC50 metrics for various chemotherapy drug or inhibitor in TCGA cohort samples. Upon analysis of 198 compounds’ sensitivity from the GDSC dataset, 99 demonstrated notably reduced IC50 levels in the low-risk cohort, in contrast to a mere two in the high-risk group. Clinically prevalent chemotherapeutic agents are detailed in **Supplementary File 1**: **Supplementary Figure S2**. Notably, individuals within the high-risk category showed increased IC50 values for agents such as 5-fluorouracil_1073, oxaliplatin_1089, camptothecin_1003, irinotecan_1088, KRAS (G12C) inhibitor-12_1885, and the VEGFR inhibitor Sorafenib_1085. (**Supplementary Figures S2C–2H**, p<0.05). Data reveal a heightened likelihood of chemotherapeutic resistance among patients designated as high-risk. However, they may exhibit sensitivity to two other drugs, specifically a small molecule inhibitor was sepantronium bromide_1941 (**Supplementary Figure S2I**, p<0.05), and non-small cell lung cancer chemotherapy drug was Vinorelbine_2048 (**Supplementary Figure S2J**, p<0.05). Therefore, these two drugs hold promise for treating colorectal cancer patients resistant to chemotherapy.

### Consensus clustering analysis

**Figure 6:**
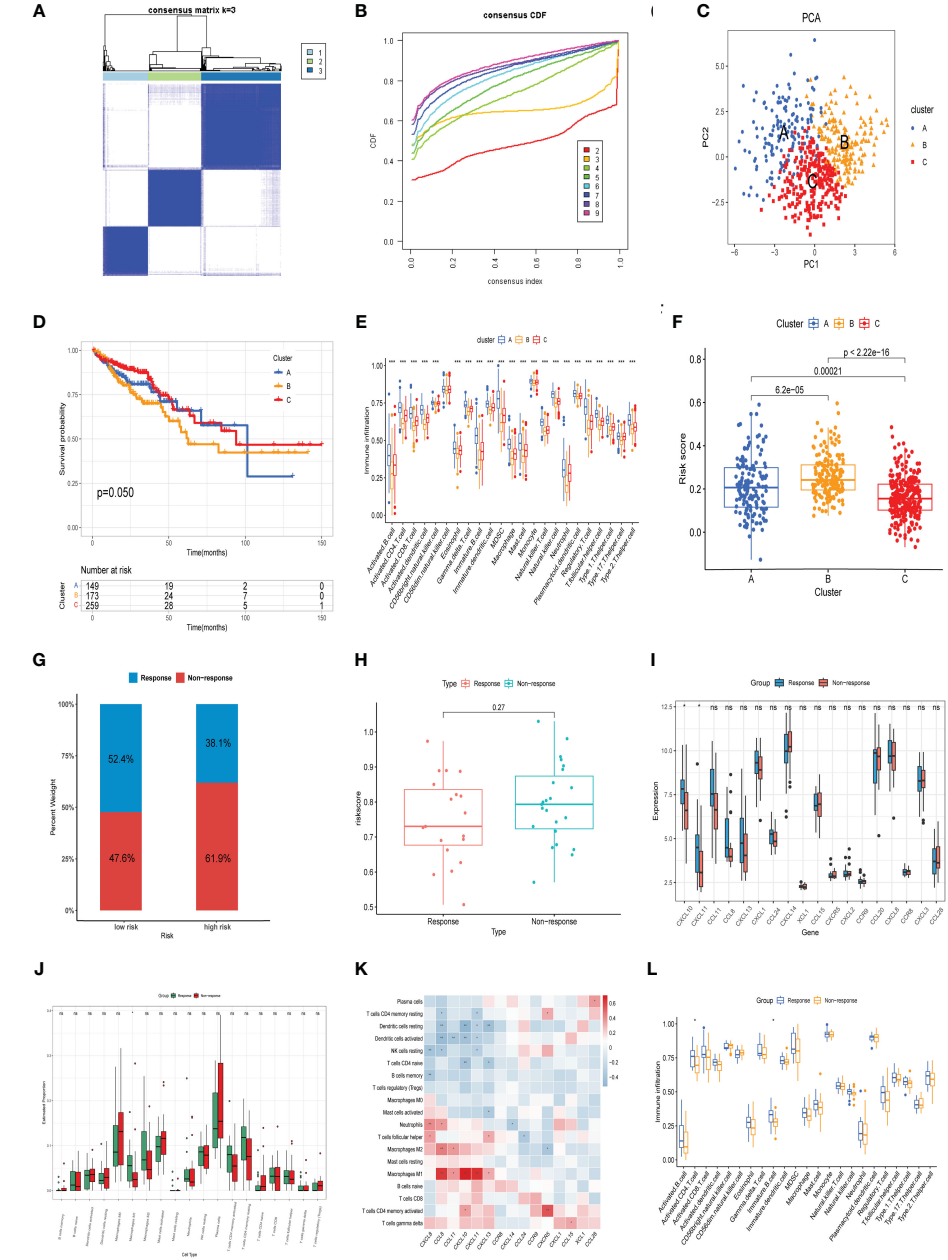
Consensus clustering analysis and neoadjuvant therapy Efficacy. **(A)** Heatmap of the consensus matrix delineating three distinct clusters (k = 3) alongside their respective areas of correlation. **(B)** Cumulative distribution function profiles for consensus clustering across a range of k values from 2 to 9. **(C)** PCA analysis showing the robustness and stability of the clustering. **(D)** Kaplan–Meier plots delineating the disparities in OS between the three clusters in TCGA cohort. **(E)** Distinct types of infiltrating immune cells in the three clusters. **(F)** Comparison of risk scores in three clusters. **(G)** The distribution of patients demonstrating a reaction to neoadjuvant treatment in the low- and high-risk groups from GSE45404 cohort. **(H)** Comparison of risk scores in response and Non response groups. **(I)** Comparison of model genes in response and Non response groups. **(J)** CIBERSORT analysis for response versus Non response groups. **(K)** Association between the expression of model genes and the prevalence of immune cells in the GSE45404 cohort. **(L)** ssGSEA analysis for response versus Non response groups. *p< 0.05, **p< 0.01, ***p< 0.001.

To better define the clinical significance and underlying biological mechanisms of these DEPCs, we performed consensus clustering analysis based on differentially expressed prognostic chemokines, the samples from the TCGA cohort were segregated into three distinct subgroups, referred to as cluster A, cluster B and cluster C. when K=3, Our analysis discerned the strongest within-group congruence and the weakest between-group associations (**Figure 6A**), which additionally provide optimal cluster robustness across a range from k=2 to k=9 (**Figure 6B**). PCA substantiated notable transcriptional distinctions across the three identified clusters (**Figure 6C**). Survival analysis indicated that individuals within Cluster C exhibited a more advantageous prognostic outcome relative to those in Clusters A and B, with Cluster B correlating with a less favorable prognosis (**Figure 6D**, p =0.050). Subsequently, ssGSEA was employed to evaluate the infiltration of various immune cells across the three Clusters, as illustrated in **Figure 6E**, it was observed that cluster B was characterized by a lack of immune cell infiltration. Finally, we compared the risk scores of patients in groups A, B, and C, these findings align with the outcomes of the survival analysis, revealing that Group B exhibited the most elevated risk score, in contrast, Group C was associated with the least risk score (**Figure 6F**).

### CXCL10 expression and M1 macrophage infiltration were associated with efficacy of neoadjuvant treatment

Evaluating the prognostic capacity of the risk score and chemokine signature-derived models in forecasting neoadjuvant treatment response, each subject within the GSE45404 cohort was assigned a risk score. Following this, stratification into low-risk and high-risk categories ensued, utilizing the median risk score as the delineation criterion. Notably, Patients within the low-risk categories demonstrated significantly enhanced efficacy in response to neoadjuvant therapy relative to their high-risk counterparts (52.4% vs.38.1%, **Figure 6G**). Additionally, non-responsive patients showed relatively higher risk score compared to responsive patients, although there was no statistical significance (**Figure 6H**). A comparison of model-based chemokine signatures between response and non-response group patients revealed indicated elevated expressions of CXCL10 and CXCL11 in the response group (**Figure 6I**). Subsequent CIBERSORT analysis indicated that the response group patients exhibited a higher infiltration of M1 macrophages (**Figure 6J**). Correlation analysis showed a significant positive linkage of CXCL10 and CXCL11 expression with M1 macrophage infiltration (**Figure 6K**). Furthermore, ssGSEA assessments indicated that patients from the response group patients manifested increased penetration of activated CD4 T cells and immature B cells (**Figure 6L**). The outcomes herein align with our prior results. In summary, the overexpression of CXCL10, activated CD4 T cells, and M1 macrophages not only reduces the risk of CRC and had a better prognosis but also enhances the efficacy of neoadjuvant therapy, suggesting their might play an essential role in antitumor immunity.

### Identification of chemokine-related prognostic genes in single−cell transcriptomics atlas

**Figure 7:**
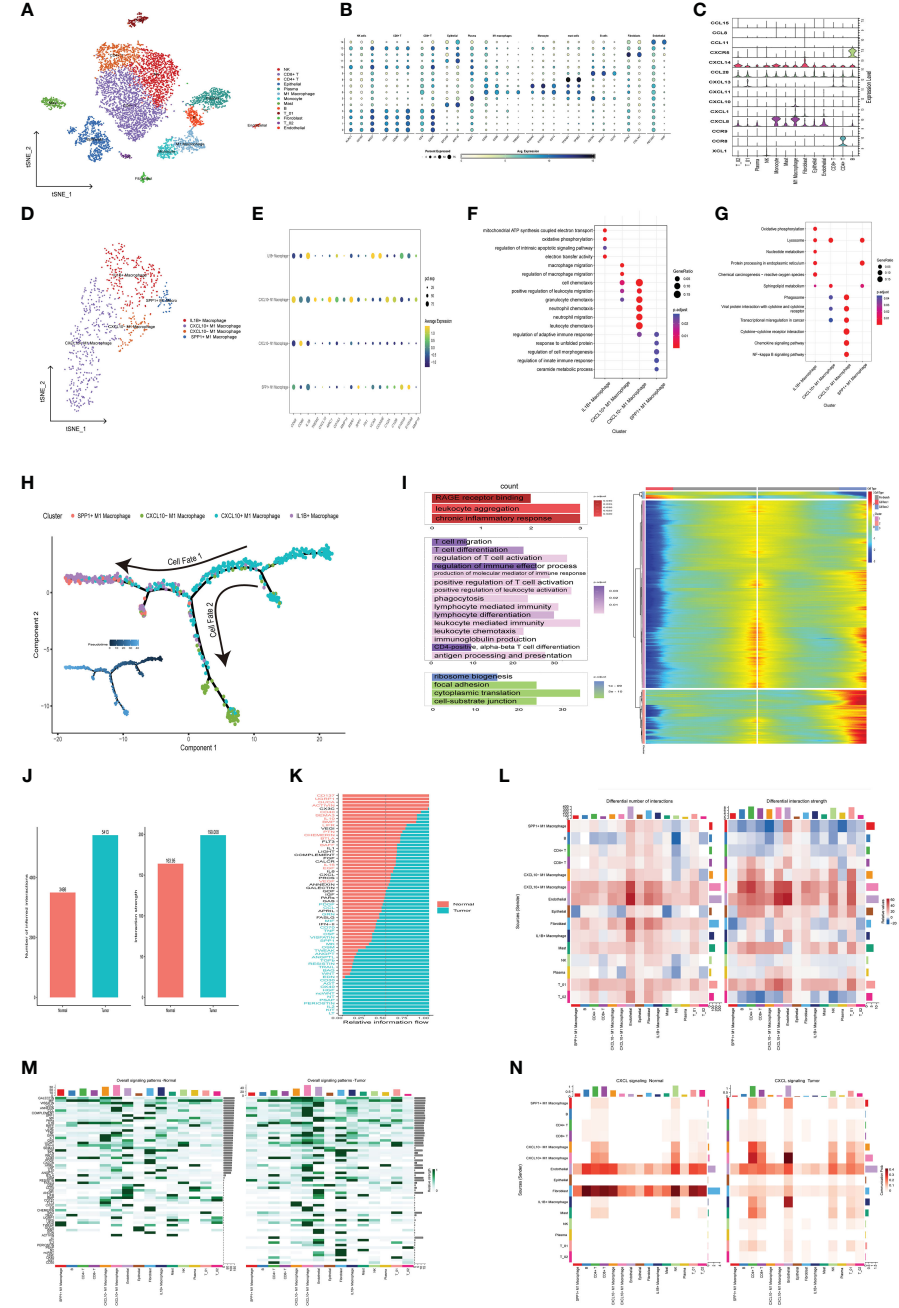
Delineation of Chemokine-Associated Prognostic Markers in Single-Cell Transcriptome Atlas. **(A)** t-SNE plot of each cluster delineated by color in accordance with established marker-defined classifications. **(B)** Bubble diagrams displaying the expression quantities of signature genes corresponding to each cellular cluster. **(C)** Violin plots of model genes expression levels for each cell cluster. **(D)** t-SNE plot of Macrophage subclusters colored by cell types. **(E)** Bubble diagrams displaying the expression quantities of signature genes corresponding to each cellular subcluster. **(F, G)** Functional enrichment of GO and KEGG leveraging differentially expressed genes in each cell **(H)** Macrophage trajectory analysis delineates two divergent cell destinies colored by cluster. The arrow indicates the likely course of evolution within the trajectory. **(I)** The heat map visualizes branching cell trajectories and gene dynamics in Macrophage cells, with columns representing cells and rows denoting genes. Genes are ranked and divided into three stages based on expression trends, accompanied by corresponding GO annotations on the left. **(J)** The bar graph represents the count and intensity of intercellular communications in Normal and Tumor. **(K)** Bar plot of tumor- or normal- specifc signaling pathways between each cell types. **(L)** Heatmaps illustrate the comparative metrics of interaction frequency (left) and relative interaction intensity (right) contrasting Normal and Tumor tissue. The upper color bar encapsulates the cumulative columnar values, indicative of incoming signals, whereas the lateral color bar aggregates the values of outgoing signals. **(M)** The series of heatmaps delineate the comprehensive signaling network activity within individual immune cell subsets, orchestrated by specific pathways in Normal (left) and Tumor (right). **(N)** The Heatmaps of CXCL signaling pathway in Normal (left) and Tumor (right).

In order to systematically assess the role of chemokine-related prognostic genes in the immune microenvironment and tumor progression of CRC, we use the GSE146771 data set, and retained a total of 7,343 cells (4,957 from tumor tissue and 2,386 from adjacent normal tissue) for subsequent analysis after quality control. We then employed the t-SNE algorithm for scRNA-seq data visualization, effectively categorizing cells into 13 distinct clusters (**Figure 7A**), Cell clusters were delineated through manual annotation leveraging established cellular markers, categorizing them into NK cells (KLRC1, CD160, NKG7), CD8+ T cells (CD3D, CD8A, GZMK), CD4+ T cells (CD3D, CD4, GZMA),Epithelial cell (EPCAM, KRT18), plasma cells (MZB1, CD79A), M1 macrophage (CD68, CD86, CD80, TREM2), Monocyte (S100A8, S100A9,CD14), Mast cells (TPSAB1, TPSB2), B cells (CD79A, MS4A1, CD19), T_01 (NKG7, GZMA), Fibroblast (ACTA2, COL1A2), T_02 (NKG7, CD8A), Endothelial (PECAM1, VWF) (**Figure 7B**). Expression of chemokine-related prognostic genes in each cell type showed in **Figure 7C**, Notably, CXCL10 expression was markedly upregulated in M1 macrophages in contrast to other cell type.

To investigate the role of CXCL10 and M1 macrophage in tumorigenesis and tumor development, we further subdivide the monocyte and M1 Macrophage cells into four subgroups based on different expressed cell markers, including IL1B+ Macrophage (CD68, CD163, IL1B), CXCL10+ M1 Macrophage (CD68, TREM2, CXCL10), CXCL10- M1 Macrophage (CD68, CD86,and low expression of CXCL10), SPP1+ M1 Macrophage (CD68, SPP1) (**Figures 7D, E**), The subsequent GO enrichment analysis suggested that IL1B+ Macrophage related to Oxidative phosphorylation (OXPHOS), regulation of intrinsic apoptotic signaling pathway, while CXCL10+ M1 Macrophage involved in macrophage migration (**Figure 7F**). Additionally, SPP1+ M1 Macrophage enriched in regulation of cell morphogenesis pathway. KEGG enrichment analysis also highlighted that IL1B+ Macrophage were significantly implicated in OXPHOS, Nucleotide metabolism and Chemical carcinogenesis − reactive oxygen species pathways (**Figure 7G**). which suggested that IL1B+ Macrophage may be implicated in the oncogenic and therapeutic resistance in colorectal cancer.

Subsequently, monocle2 was utilized to infer the potential developmental trajectory of Macrophage cells. Remarkably, we observed a well-ordered differentiation pattern wherein CXCL10+ M1 Macrophages were predominantly situated at the initial point of the developmental trajectory, while CXCL10- M1 Macrophages, IL1B+ Macrophages and SPP1+ M1 Macrophages were located at separate branches (**Figure 7H**). Further, branched expression analysis modeling (BEAM) indicated that 848 genes with significant expression changes, which could be clustered into three expression patterns: Cell fate 1 were associated with chronic inflammatory response. The pre-branch cluster were involved in the positive regulation of T cell activation, production of molecular mediator of immune response and leukocyte mediated immunity pathway. The Cell fate 2 were enriched in focal adhesion and cell−substrate junction (**Figure 7I**). In summary, CXCL10+ M1 macrophages may promote the activation and differentiation of T cells to enhance anti-tumor immunity.

### Intercellular interaction in the TME

To assess the prospective interplay within the tumor microenvironment, we utilized CellChat analysis to establish divergent networks of intercellular communication within tumors and normal tissues. we observed that numbers of interactions and strength of interactions were markedly elevated in the tumor group than in normal group (**Figure 7J**). An integrative analysis of communication likelihoods within the information network was conducted to discern the differential in cumulative information flux between the control and tumor cohorts. Findings elucidated an elevated presence of CD137, ACTIVIN, CD40, IL10, BAFF, IL16, EGF, and VEGF signaling pathways in normal group (red), in contrast to an augmented prevalence of kIT, IL2, WNT, TGFb, CCL, GRN, MIF, TNF, CSF, and SPP1 signaling pathways within the tumor group (green) (**Figure 7K**). Remarkably, outgoing signals from CXCL10+ M1 Macrophages to B cells, CD4+T cell, CD8+T cells and Endothelial cell were more abundant and stronger in tumor group compared with normal group, while the output signal of SPP1+ macrophages cell was reduced in the tumor group (**Figure 7L**). The visualization results of overall information flow revealed that the information flow from CXCL10+ M1 macrophages and CD4+T cell was significantly increased in the tumor group (**Figure 7M**). Notably, CXCL signaling network exhibit heightened activity in mediating communication between CXCL10+ M1 macrophages and CD4+T cell, with significantly elevated activity within the tumor group compared to the normal group (**Figure 7N**). Further analysis reveals that communication through CXCL16-CXCR6 pairs between these cells is also markedly higher in the tumor group (**Supplementary Figure S2K**). Additionally, studies report that CXCL16-CXCR6 pairs enhance T cell positioning and differentiation, particularly into resident memory T cells, thereby improving tumor immunosurveillance and therapeutic efficacy (19). In conclusion, CXCL10+ M1 macrophages are pivotal in modulating cell-to-cell communication in CRC and hold potential as a strategic avenue for CRC therapy.

### Single-cell expression atlas of neoadjuvant immunotherapy-treated CRC and variations in cancer stemness among diverse therapeutic outcomes

**Figure 8:**
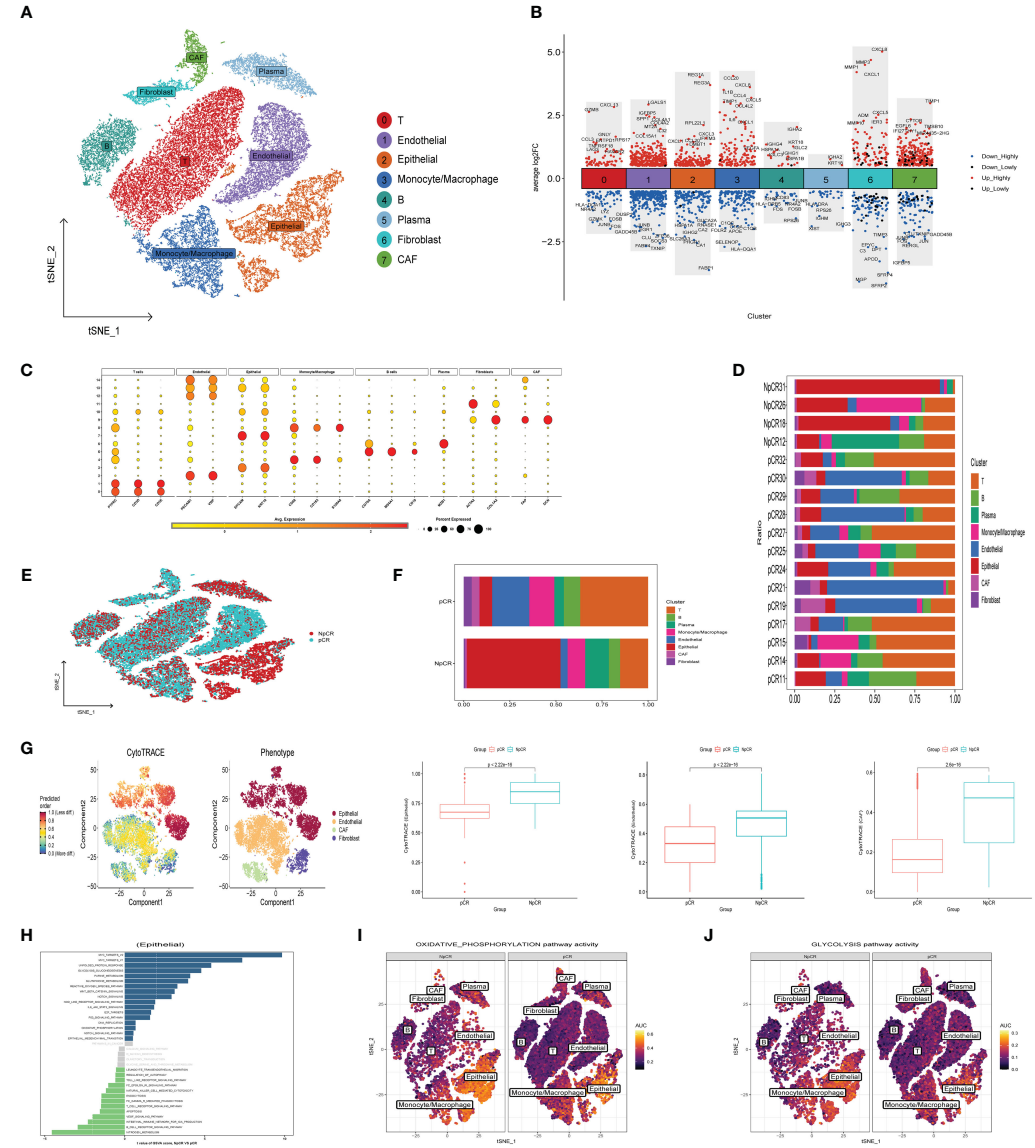
Landscape of neoadjuvant immunotherapy-treated CRC and Cancer Stemness. **(A)** t-SNE plot of each cluster delineated by color in accordance with established marker-defined classifications. **(B)** DEGs that differentiate the NpCR group from the pCR group were detected across diverse cell types. **(C)** Bubble charts representing the expression of signature genes within respective cellular clusters. **(D)** Bar graphs displaying the relative abundance of major cellular populations across individual patients. **(E)** t-SNE visualization of the cellular landscape categorized by NpCR and pCR across distinct major cell populations. **(F)** proportion of major cell types in NpCR group and pCR group. **(G)** tSNE diagrams display the variance in CytoTRACE evaluations among four distinct cell categories. The coloration transitions from dark green, denoting lesser values indicative of reduced stemness, to dark red, representing greater values associated with heightened stemness. and compare the stemness scores between NpCR group and pCR group. **(H)** Variations in intracellular pathway activations, quantified through GSVA, contrasting NpCR and pCR group **(I, J)** Overlay of single-cell AUC evaluations corresponding to activities within the OXPHOS and glycolysis pathways, respectively.

To elucidate disparities in the efficacy of neoadjuvant immunotherapy, a total of 37287 cells (8812 from non-pathological complete response samples and 28475 from pathological complete response samples) from 17 tumor tissue were analyzed after quality control in our study. Subsequently, the t-SNE dimension reduction method was employed to successfully identify 8 major cell subsets according to typical marker genes (**Figure 8A**), including T cells (PTPRC, CD3D, CD3E), Endothelial cells (PCAM1, VWF), Epithelial cells (EPCAM, KRT18), Monocyte/Macrophage (CD68, CD163, S100A8), B cells (CD79A, CD19B, MS4A1), Plasma cells (MZB1), Fibroblasts (ACTA2, COL1A2), CAF (FAP, DCN) (**Figure 8C**). Moreover, **Figure 8B** illustrates the differential genes across cell subgroups within the NpCR and pCR groups. In the NpCR group, elevated expression of immunosuppressive receptors TNFRSF18, LAG3, and HAVCR2 in the T cell subpopulation, as well as elevated levels of pro-inflammatory chemokines (CCL20, CXCL8, CCL4) and cytokines (IL1B, IL6) in Monocyte/Macrophage subsets identified within the NpCR group. However, in the pCR group, HLA-DQA1 was found to be upregulated in T cells subsets, and complement genes C1QA, C1QB, and C1QC were upregulated in Monocyte/Macrophage subsets, while HLA-DRB5 and HLA-DRA exhibited increased expression in B cells and plasma cells subsets, respectively. To summarize, the upregulation of antigen-presenting HLA genes, activation of the complement cascade, and inhibition of IL1B+ monocyte mediated pro-inflammatory response may enhance the efficacy of neoadjuvant therapy (14).

To reveal the difference of TME remodeling between the two therapeutic effects, we compared the cell composition between each patient (**Figure 8D**) and different response groups (**Figures 8E, F**). Increased presence of T cells, B cells, and monocyte/macrophage subsets was observed in the pCR group, in contrast to the notably elevated epithelial cells in the NpCR group. Additionally, to explore the correlation between tumor stemness attributes and outcomes of neoadjuvant therapy, we employed the CytoTRACE algorithm to calculate stemness scores. We observed that epithelial cells, endothelial cells and CAF cells stemness scores were significantly increased in the NpCR group, suggesting that higher differentiation potential was associated with worse neoadjuvant efficacy (**Figure 8G**). The GSVA results presented in **Figure 8H** indicate a marked enrichment in pathways associated with tumor proliferation and metastasis in the NpCR group, including MYC targets, Wnt β catenin, EMT, and purine metabolism pathways. Moreover, the NpCR group showed increased activities in OXPHOS and glycolytic metabolism pathways (**Figures 8I, J**). Conversely, pCR group associated with anti-tumor immunity, including B cell receptor signaling, natural killer cell mediated cytotoxicity and apoptosis pathway.

### Heterogeneity of macrophages between different therapeutic effects

**Figure 9:**
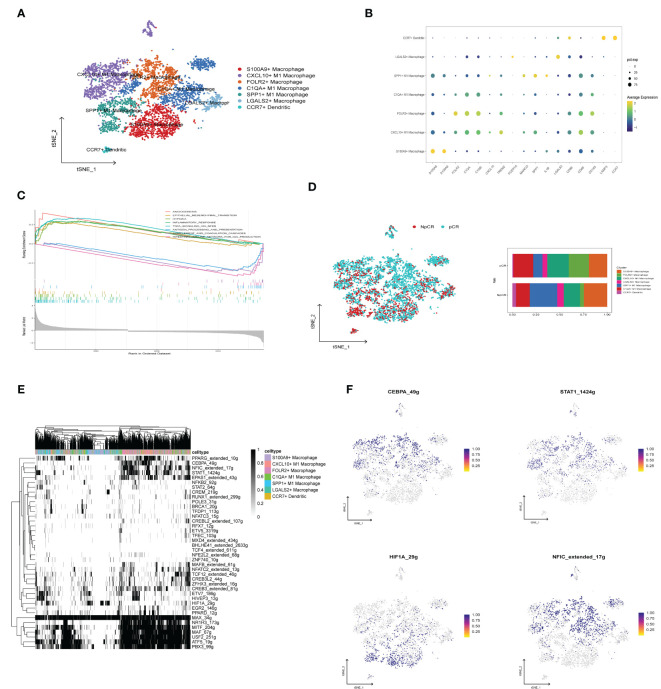
Evaluation and Validation of chemokine Related Prognostic Model. **(A)** t-SNE plot of monocyte subclusters colored by cell types. **(B)** Bubble charts representing the expression of signature genes within respective cellular clusters. **(C)** GSEA analysis between NpCR group and pCR group in monocyte subclusters. **(D)** t-SNE plot of all cells colored by NpCR group and pCR group in monocyte subclusters and proportion of monocyte subclusters in NpCR group and pCR group. **(E)** Activity of cell-specific regulons was encoded in a binary matrix: exceeding a custom-set AUC threshold denoted activation (black), while values beneath indicated inactivity (white). **(F)** tSNE visualizations represent the distribution of transcription factor expression levels, delineated by binarized activity based on AUC scores.

To investigate the heterogeneity of macrophages among different therapeutic effects, we further subdivided the monocyte and macrophage cells into seven subgroups based on differentially expressed cell markers (**Figure 9A**), including S100A9+ Macrophage (CD68, S100A9, S100A8), CXCL10+ M1 Macrophage (CD68, TREM2, CXCL10), FOLR2+ Macrophage (CD68, FOLR2, TREM2), C1QA+M1 Macrophage (CD68, C1QA, C1QB), SPP1+ M1 Macrophage (CD68, SPP1), LAGALS2+ Macrophage (CD68, LAGALS2, FCER1A), CCR7+ Dendritic (LAMP3, CCR7) (**Figure 9B**). The GSEA findings revealed that angiogenesis, EMT, hypoxia, and inflammatory response pathways were enriched by the upregulated genes within the macrophage subpopulation of the NpCR group, whereas the pCR group related to intestinal immune network for IgA production, complement and coagulation cascades, and antigen processing and presentation pathways (**Figure 9C**). Furthermore, the pCR group exhibited a higher proportion of S100A9+ Macrophage, CXCL10+ M1 Macrophage, and FOLR2+ Macrophage relative to NpCR group (**Figure 9D**). Moreover, we utilized SCENIC to assess the upstream transcription factors driving heterogeneity of macrophages. Our investigation revealed distinct activity profiles of pivotal transcription factors (TFs) across macrophage subpopulations. Specifically, the transcription factor CEBPA, a promoter of myeloid cell differentiation (20), STAT1, an inhibitor of stemness properties and cell proliferation (21), and NFIC, The TF of has been reported to inhibit pancreatic cancer (22), were found to be substantially upregulated in the CXCL10+ M1 and FOLR2+ M1 macrophages, which demonstrated increased cell counts within the pCR group. Simultaneously, we identified a pronounced expression of the TF HIF1A, known to facilitate tumor immune escape (23) within SPP1+ M1 macrophages, a population that was notably abundant in the NpCR group (**Figures 9E, F**).

### Experimental validation

**Figure 10:**
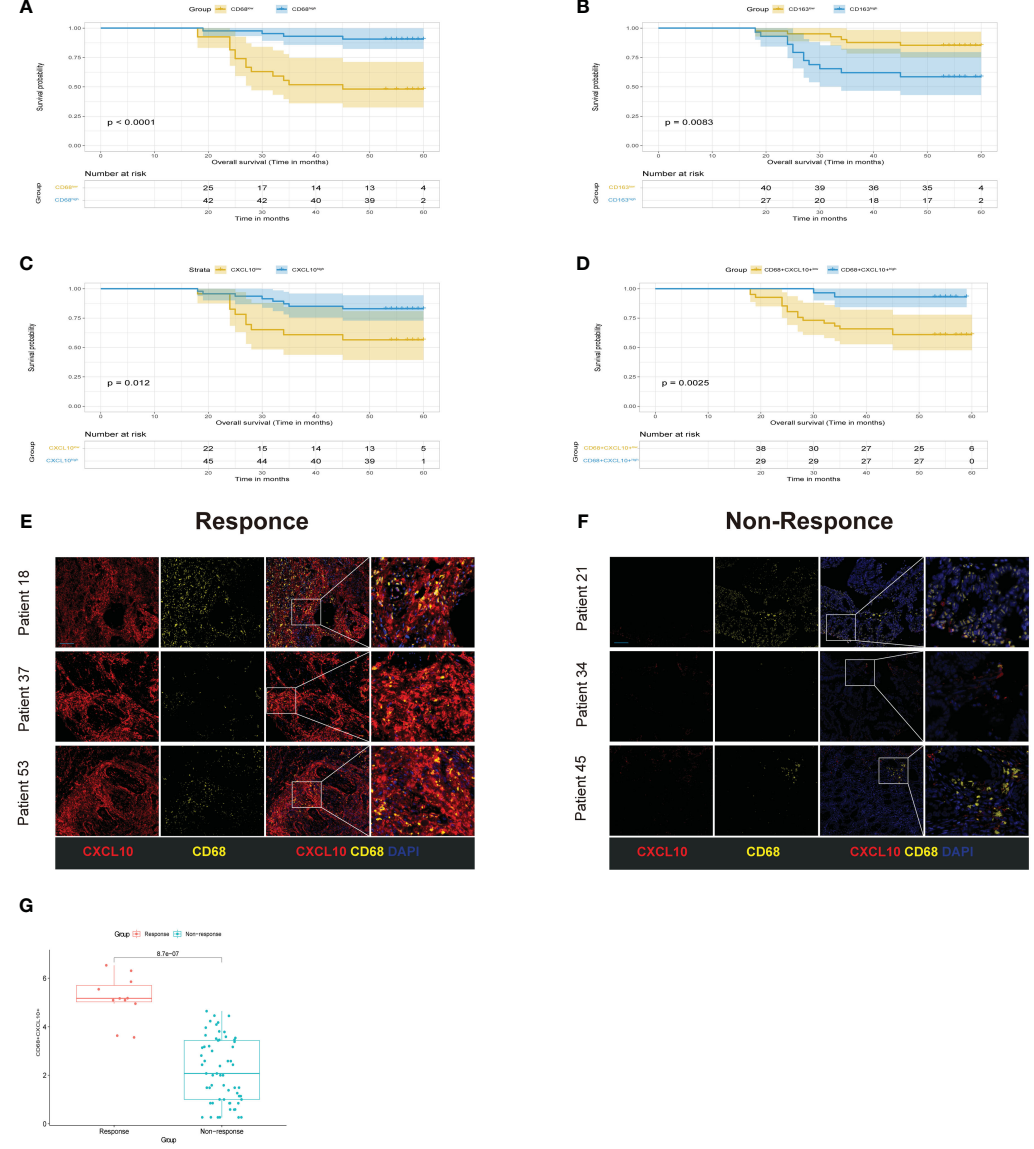
Experimental validation. **(A-D)** Kaplan-Meier curve of OS for CRC patients grouped by the expression levels. **(E, F)** Multiplex immunofluorescence images of different cell populations in Response group and Non-response group. CXCL10(red), CD68 (yellow), DAPI (blue), scale bar=100µm. **(G)** compare the expression of CXCL10+CD68+ cells between Response group and Non-response.

To corroborate our initial observations, we conducted immunofluorescence staining on tissue specimens from cohort 1. The Kaplan-Meier survival diagrams underscored a notable correlation between the heightened concentration of the M1 macrophage marker CD68, the decreased presence of the M2 macrophage marker CD163, and increased expression of CXCL10. These findings were particularly evident in CRC samples with favorable prognostic outcomes (**Figures 10A–C**). Notably, samples with high expression of both CXCL10 and CD68 also showed a significant survival advantage (**Figure 10D**). In order to further explore the possibility of the signature from CXCL10+CD68+ cells operating as an advantageous biomarker for neoadjuvant therapy. We also performed mIHC staining in cohort 2, This cohort comprised 72 CRC patients post-neoadjuvant therapy, with 11 patients exhibiting complete or near-complete pathological response (TRG0 or TRG1) classified as the Response group, and 61 patients with minimal or no response (TRG2 or TRG3) as the Non-Response group. The findings demonstrated a pronounced increase in the frequency of CXCL10+CD68+ cells in the specimens from the responders relative to those from the non-responders (**Figures 10E–G**). This is consistent with our previous conclusions.

## Discussion

Despite substantial advancements in the fields of molecular biology and immunology lately, the prognosis for patients with advanced colorectal cancer continues to be disheartening. Moreover, therapeutic opportunities continue to be limited for patients without identifiable drug targets. Immunological checkpoint blockade (ICB) therapy has achieved significant breakthroughs in treating advanced malignancies, offering enduring therapeutic outcomes for a subset of patients. However, it is disappointing that immunotherapy is only efficacious for a small number of patients with MSI-H, and those who initially respond well may subsequently develop resistance. Moreover, the concurrent use of specific drugs holds potential for enhancing therapeutic efficacy and mitigating related toxicity. The current emphasis is on identifying a multitude of biomarkers and establishing standardized scoring systems for patient stratification, with the aim of benefiting a wider population. For the majority of colorectal cancer patients characterized by MSS, ICB monotherapy has proven to be ineffective. Beyond the status of microsatellites, the development of other potential biomarkers could help identify patient groups that may derive benefit (24, 25). Chemokines play an indispensable role in guiding immune cell migration, a vital process for instigating and subsequently implementing a powerful immune response against tumors. Additionally, Chemokines and their receptors hold considerable value as biomarkers for cancer forecasting (7). However, there remains a distinct lack of comprehensive research exploring the role of chemokines in CRC.

In our research, we devised a risk score derived from 15 Chemokines and identified the risk score as an independent prognostic element. Furthermore, we confirmed a marked linkage between the risk score and clinical pathological factors. Subsequently, we stratified patients from the TCGA and GSE39582 datasets into dual categories rooted in the risk score and confirming that patients possessing a low-risk score exhibited a significant survival advantage. In an effort to enhance the accuracy of prognosis, we integrated indicators such as T stage, risk score, age, and pathological stage to construct a nomogram. Validation using the external GSE39582 dataset substantiated its superior predictive capability and clinical utility compared to traditional TNM staging. The establishment of this model offers an innovative and efficacious instrument for forecasting the clinical outcome of CRC patients. To explore the potential processes explaining the varied results among categories with high and low risk, we conducted an enrichment analysis. GSEA outcomes demonstrated a substantial enrichment of the EMT pathway in the high-risk groups within both TCGA and GSE39582 datasets. This pathway is known to significantly influence tumor advancement, metastasis, and drug insusceptibility in CRC (26). Furthermore, the high-risk population also exhibited enrichment in the hypoxia pathway, which is intimately linked with enhanced drug insusceptibility and remote metastasis in CRC (27). The mTORC1 signaling pathway was also found to be enriched, a pathway previously reported to foster tumor growth and limit therapeutic response (28). These pathways hold substantial potential as targets for drug-resistant treatments in colorectal cancer. In addition, In the high-risk category, there was a notable upregulation of PDCD1 and CD274, suggesting that subjects within this group could potentially benefit from anti PD-1/PD-L1 treatment. A comprehensive understanding of the tumor TME can help identify novel approaches for treating CRC. Therefore, high-risk and low-risk populations were subjected to an analysis with the application of CIBERSORT algorithms to assess differences in the composition of immune cellular infiltrates. The CIBERSORT assessment revealed an increased proportion of CD4+ T cells within the low-risk patients, whereas a pronounced abundance of M2 macrophages and Treg cells characterized the high-risk. CD4+ T cells strengthen antitumor defenses through the efficacy of cytotoxic T lymphocytes (29). Conversely, Treg cells primarily suppress antitumor immune responses in the tumor tissue by expressing inhibitory molecules (30). Moreover, a notable correlation exists between the infiltration of M2 macrophages and both the proliferation and adverse clinical outcome in CRC (31). This observation could potentially explain the propensity of high-risk group patients to exhibit drug resistance and unfavorable prognostic outcomes. An analysis comparing drug sensitivity was performed across high-risk and low-risk categories. The results revealed that the high-risk group exhibited a heightened IC50 for standard chemotherapeutic drugs used in the treatment of CRC, including 5-fluorouracil, oxaliplatin, camptothecin, and the Irinotecan. This discovery might offer additional insights into the heightened tendency for drug resistance observed among the high-risk group patient.

The implementation of neoadjuvant therapy strategies has notably amplified the rate of tumor reduction before surgery and lessened the occurrence of local and regional relapses in subjects with locally advanced rectal carcinoma. Nevertheless, the risk of distant metastasis remains high, and there are numerous uncertainties regarding the optimal selection of beneficiaries, choice of the most effective treatment strategies, and identification of valid biomarkers. Consequently, there is a critical need to develop more accurate and dependable predictive biomarkers for the identification of patients likely to respond favorably to neoadjuvant treatments (32). On this basis, we further explored the differential expression of chemokine-related gene signature in different treatment outcomes. It is noteworthy that patients categorized within the low-risk populations exhibited a markedly enhanced response to neoadjuvant therapy relative to their high-risk counterparts, demonstrating a considerable increase in treatment effectiveness. Furthermore, the chemokines CXCL10, CXCL11, and M1 macrophages were highly expressed in response group. Previous reports have indicated that the axis comprising CXCL9, CXCL10, and CXCL11 alongside CXCR3 is pivotal in directing the recruitment, maturation, and stimulation of immune cell populations. This signaling pathway is central to immune responses, modulating the maturation of naïve T cells into T-helper 1 phenotype while concurrently directing immune cell populations to specifically migrate to the sites of lesions, thereby exerting their immunological functions (33). Investigations revealed that heightened CXCL10 expression impedes tumor proliferation while enhancing CD8+ T cell penetration, thereby facilitating the normalization of tumor vasculature. This phenomenon renders colorectal cancer cells increases the responsiveness of the synergistic effects of cetuximab and anti-PD1 treatment (34). Therefore, we postulate that CXCL10 could act as a prospective indicator for the effectiveness of neoadjuvant therapy.

Single-cell sequencing is a powerful tool that offers many advantages compared to traditional bulk RNA-seq methods, enabling analysis at the single-cell resolution level and significantly enhancing data quality. This has already exerted influence on numerous fields of oncology research, bolstering our insight into tumor diversity and TME (35). In this study, we characterized the colorectal cancer landscape using single-cell data and discovered that CXCL10 is highly expressed in M1 macrophages. Through the utilization of CellChat analysis, we observed that outgoing signals from CXCL10+ M1 macrophages to B cells, CD4+ T cells, CD8+ T cells was noticeably enhanced within the tumor group. This observation is potentially linked to the role of CXCL10-positive M1 macrophages in recruiting immune cells within tumor tissues. Following this, upon evaluating scRNA data from patients after undergoing neoadjuvant therapy, we detected a considerable augmentation in the epithelial cell population within the NpCR group relative to the pCR group. By calculating the stemness score, our analysis revealed that the stemness score associated with the NpCR group was significantly elevated relative to the pCR group. A study, validated across multiple datasets, revealed a correlation between tumor stemness and resistance to immune checkpoint inhibitor (ICI) treatment (36), which is consistent with our findings. Furthermore, we noted an increase in the activity of OXPHOS and glycolytic metabolic pathways in epithelial cells within the NpCR group. The Warburg effect, as described in a study, suggests that even well-oxygenated cancer cells undergo glycolysis, leading to the prevalent hypothesis of downregulated OXPHOS in cancer. However, recent research indicates that OXPHOS can also be upregulated in certain types of cancer. Therefore, Inhibitors of OXPHOS might be strategically harnessed to address tumor subtypes that display an upregulation of OXPHOS (37). Finally, through further subclustering of mononuclear cells, we identified several new subgroups and observed an increase in FOLR2+ macrophages and CXCL10+ M1 macrophages within the pCR group, whereas the NpCR cluster witnessed an upswing in SPP1+ macrophages. Recent research reported a notable association between the abundance of FOLR2+ macrophages and improved prognostic outcomes in individuals diagnosed with breast cancer. Additionally, it has been observed that the occurrence of these FOLR2+ macrophages is found to be associated with an increased prevalence of CD8+ T cells, suggesting an anti-tumor immunological function (38). Literature suggests that the chemotactic draw of effector T cells into tumor sites, orchestrated by CXCL9 and CXCL10, aids in the establishment of a “hot” TME characterized by T cell inflammation. M1 macrophages and dendritic cells undertake a significant function in the profuse creation of CXCL9 and CXCL10.Therefore, their manifestation in the TME becomes a vital prerequisite for T cell infiltration, and it facilitates the structuring of the T cell inflammatory TME. In an effort to amplify the efficacy of immunotherapies, it becomes necessary to adopt therapeutic interventions that promote the recruitment of bone marrow cells capable of producing these chemokines, while also activating the innate immune pathways they initiate. Such strategies hold potential for widespread clinical applications (39). Research findings suggest that FAP+ fibroblasts and SPP1+ macrophages are involved in the restructuring of the extracellular matrix and aid in establishing a pro-fibrotic stromal microenvironment, This action obstructs lymphocyte entry into the tumor core, thereby further curtailing the effectiveness of PD-L1 therapy (40). Finally, through multiple immunofluorescence experiments, we have confirmed the elevated presence of CXCL10+M1 macrophages in tissue samples from patients who responded favorably to neoadjuvant therapy, and we have also discovered a significant association between the heightened expression of CXCL10+M1 macrophages and superior prognosis. Concurrently, this insight implies that these chemokines might have a crucial role in the anti-tumor immunity of CRC, thereby offering a novel research direction for future CRC investigations.

Even though our research has made notable advancements, it’s essential to be aware of its limitations. Primarily, our investigation utilizes a retrospective approach to analyze public databases, and potential sample selection bias may impact the accuracy of the study results. To validate these findings, it is essential to conduct comprehensive prospective investigations, supplemented by extensive experimental work to strengthen our evidence. Secondly, although our study primarily relies on transcriptomic data, probing into proteomics and spatial transcriptomic data undeniably carries substantial importance for a holistic insight into the tumor microenvironment and the intricate dynamics of tumorigenesis.

## Conclusion

To conclude, our research has developed a solid prognostic model predicated on chemokines. utilizing a comprehensive analysis of single-cell and aggregate RNA transcriptomic data, We have efficaciously pinpointed CXCL10+ M1 macrophages as a predictive bioindicator for gauging the effectiveness of neoadjuvant therapy. This pivotal discovery could stand as a new clinical bio-indicator, proficient not just in foretelling the prognosis of CRC patients and their response to neoadjuvant therapy, but also in facilitating the development of more precise personalized treatment strategies.

## Data availability statement

Publicly available datasets were analyzed in this study. This data can be found hereUCSC, https://xenabrowser.net/datapages/ https://www.ncbi.nlm.nih.gov/geo TCGA-COAD, TCGA-READ, GSE39582, GSE146771, GSE205506.

## Ethics statement

The studies involving humans were approved by ethics committee of the Ruijin Hospital. The studies were conducted in accordance with the local legislation and institutional requirements. Written informed consent for participation in this study was provided by the participants’ legal guardians/next of kin.

## Author contributions

AT: Conceptualization, Validation, Visualization, Writing – original draft. JH: Data curation, Formal analysis, Methodology, Writing – original draft. ZL: Data curation, Methodology, Validation, Writing – original draft. YZ: Conceptualization, Data curation, Writing – review & editing. FC: Investigation, Writing – review & editing. JZ: Conceptualization, Funding acquisition, Methodology, Resources, Supervision, Writing – review & editing. WF: Data curation, Investigation, Writing – review & editing. ZX: Conceptualization, Investigation, Writing – review & editing. ZM: Conceptualization, Resources, Writing – review & editing. PX: Methodology, Resources, Supervision, Writing – review & editing. AL: Investigation, Methodology, Resources, Supervision, Writing – review & editing.
