# Supplementary material for: Establishment of a chemokine-based prognostic model and identification of CXCL10+ M1 macrophages as predictors of neoadjuvant therapy efficacy in colorectal cancer
Source: Front Immunol. 2024 Aug 7;15:1400722. doi: 10.3389/fimmu.2024.1400722 (PMC11335547; doi:10.3389/fimmu.2024.1400722)
Supplement: Supplementary file 1 [file DataSheet_1.docx]

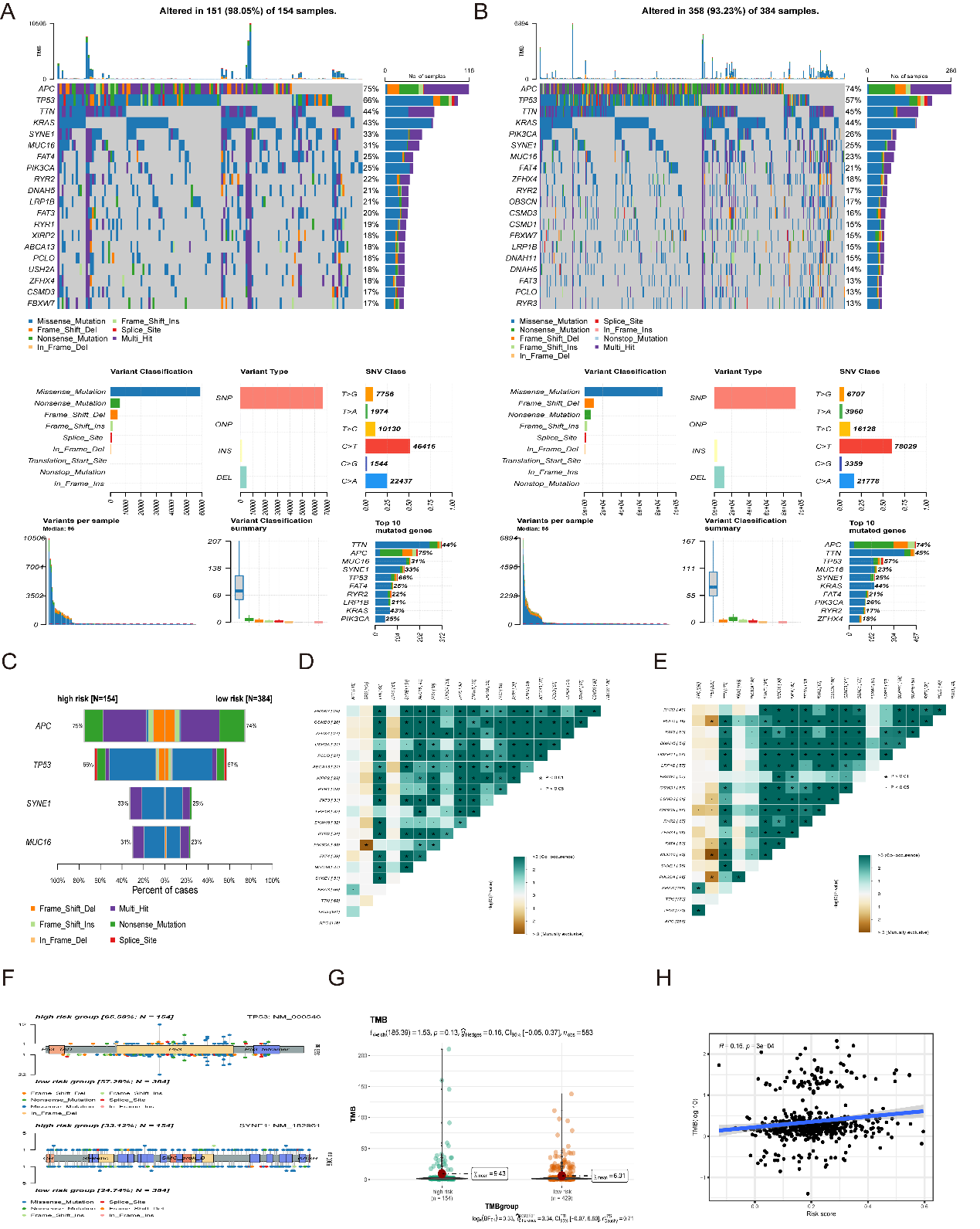
.

Supplementary Figure 1. Evaluation and Validation of chemokine Related Prognostic Model.

(A-C) Oncoplots and summary plots illustrate the somatic mutation disparity between high and low-risk groups in the TCGA cohort. (D, E) Heatmap displays the correlation of co-occurring and mutually exclusive mutations in groups with varying low-risk groups (axes denote genes; colors indicate correlations: green for co-occurring, brown for mutually exclusive). (F) Diagram depicts highly mutated domains in TP53 and SYNE1 protein structures (lollipop colors signify somatic mutation types). (G) Boxplot represents signature tumor mutation burden variation between risk groups. (H) Association between risk score and TMB (log 10) is shown.


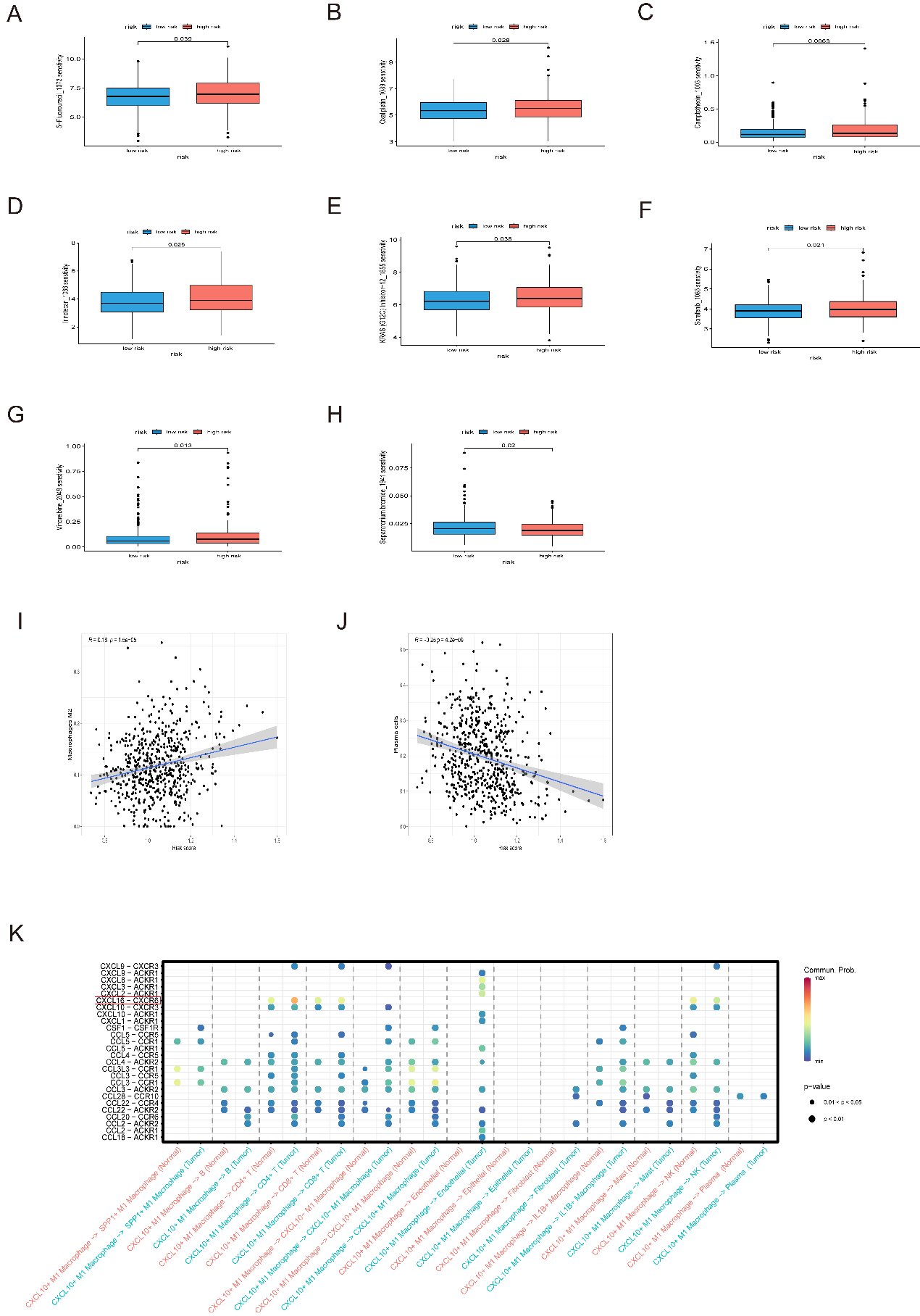


Supplementary Figure 2. Drug sensitivity and Correlation analysis

(A-H) Boxplots illustrate the average discrepancies in the estimated IC50 values of eight representative drugs (5-fluorouracil_1073, oxaliplatin_1089, camptothecin_1003, Irinotecan_1088, KRAS (G12C) inhibitor -12_1885, Sorafenib_1085, sepantronium bromide_1941, and Vinorelbine_2048). (I) The association between risk score and M2 macrophage infiltration. (J) The association between risk score and plasma cell infiltration. (K) The bubble plots illustrate potential ligand-receptor interactions between macrophage subpopulations and other cell subgroups, comparing interaction probabilities between tumor and normal groups.

Supplementary Table 1

|  |  | TCGA cohort | GSE39582 | p |
| --- | --- | --- | --- | --- |
|  |  | n=581 | n=556 |  |
| age (mean (SD)) |  | 65.65 (12.42%) | 66.73 (13.29%) | 0.158 |
| gender (%) | Female | 264 (45.4%) | 249 (44.8%) | 0.871 |
|  | male | 317 (54.6%) | 307 (55.2) |  |
| T (%) | T0 | 1 (0.2%) | 4 (0.7%) | <0.001 |
|  | T1 | 19 (3.3%) | 11 (2.0%) |  |
|  | T2 | 103 (17.7%) | 44 (7.9%) |  |
|  | T3 | 395 (68.0%) | 360 (64.7%) |  |
|  | T4 | 62 (10.7%) | 117 (21.0%) |  |
|  | N/A | 1 (0.2%) | 20 (3.6%) |  |
| N (%) | N0 | 328 (56.5%) | 295 (53.1%) | <0.001 |
|  | N1 | 144 (24.8%) | 131 (23.6%) |  |
|  | N2 | 106 (18.2%) | 98 (17.6%) |  |
|  | N3 | 0 (0.0%) | 6 (1.1%) |  |
|  | N/A | 3 (0.5%) | 26 (4.7%) |  |
| M (%) | M0 | 433 (74.5%) | 474 (85.3%) | <0.001 |
|  | M1 | 83 (14.3%) | 60 (10.8%) |  |
|  | N/A | 65 (11.2%) | 22 (4.0%) |  |
| stage (%) | 0 | 0 (0.0%) | 4 (0.7%) | <0.001 |
|  | I | 102 (17.6%) | 32 (5.8%) |  |
|  | II | 207 (35.6%) | 258 (46.4%) |  |
|  | III | 168 (28.9%) | 203 (36.5%) |  |
|  | IV | 84 (14.5%) | 59 (10.6%) |  |
|  | N/A | 20 (3.4%) | 0 (0.0%) |  |
| status (%) | Alive | 462 (79.5%) | 369 (66.4%) | <0.001 |
|  | Dead | 119 (20.5%) | 187 (33.6%) |  |
